# Development of Advanced Oral-on-a-Chip: Replicating the Intricate Human Oral Microenvironment

**DOI:** 10.7150/ijbs.104351

**Published:** 2024-10-28

**Authors:** Soo-Rim Kim, Eun-Kyung Min, Choon-Mi Lee, Jin Woo Lee, Chan Hum Park, YunJae Jung, Byung-Chul Oh, Hwa-Yong Lee

**Affiliations:** 1 Department of Health Sciences and Technology, GAIHST, Gachon University, Incheon, 21999, Republic of Korea.; 2 Department of Molecular Medicine, School of Medicine, Gachon University, Incheon 406-840, Republic of Korea.; 3 Department of Otolaryngology-Head and Neck Surgery, Chuncheon Sacred Heart Hospital, Hallym University College of Medicine, Chuncheon, Republic of Korea.; 4 Department of Microbiology, College of Medicine, Gachon University, Incheon 21999, Republic of Korea.; 5 Department of Physiology, Lee Gil Ya Cancer and Diabetes Institute, Gachon University College of Medicine, Incheon, 21999, Republic of Korea.; 6 Division of Science Education, Kangwon National University, 24341, Republic of Korea.

**Keywords:** Tonsil-resident stem cells, Natural polymers, Oral on a chip, Toxicity screening, SERPINB2

## Abstract

The interactions between various cellular populations in the oral cavity, including gingival keratinocytes, tonsil-resident stem cells, periodontal ligament fibroblasts, and vascular endothelial cells, are crucial for maintaining oral health. These interactions regulate essential functions like tooth support and pathogen defense. However, conventional 2D and 3D *in vitro* models often fail to capture the complexity of these interactions and the multicellular architecture of the oral environment. To address this limitation, we developed an advanced 3D oral-on-a-chip system that mimics the dynamic microenvironment of oral tissues. This system incorporates multiple oral cells into a 3D structure made from natural polymers such as collagen and hyaluronic acid, crosslinked by blood-coagulating factors. Our study revealed that tonsil-resident stem cells are more sensitive to toxic exposure compared to differentiated cells like fibroblasts and endothelial cells. SERPINB2 was identified as a key biomarker of oral toxicity, with significant upregulation observed in tonsil-resident stem cells after exposure to toxins. Based on this, we developed a fluorescence-linked toxicity detection system using SERPINB2, enabling sensitive and quantitative assessments of oral toxicity. This integrated system provides a valuable tool for evaluating the oral toxicity of drug candidates.

## Introduction

Due to significant physiological and genetic differences between species, animal models often fail to reflect human responses accurately 1. This can lead to unreliable outcomes and poor extrapolation to human conditions 1. However, traditional single-cell monolayer culture systems lack the complex cell-cell and cell-matrix interactions found in tissues, resulting in oversimplified models that cannot accurately represent the 3D tissue microenvironments and functional dynamics of oral tissues 2. To address these significant limitations, many innovative oral-on-a-chip systems are being developed to more closely replicate human physiological conditions in three-dimensional (3D) tissue structures 3-5. While notable, these 3D cell culture methodologies are limited in fully replicating the fundamental structure of oral tissues and the detailed, mutual cellular interactions among diverse oral cellular components that are crucial in sustaining oral homeostasis and functions 5-7. To develop a multi-chamber oral-on-a-chip model utilizing polydimethylsiloxane (PDMS), we employed computer-aided design (CAD) and 3D printing methods to create a compartmentalized microchannel layout consisting of stem cells, including tonsil, periodontal ligament, and gingival keratinocyte chambers, separated by a thin porous barrier. In addition, to accurately replicate the 3D oral microenvironment, various oral constituent cells were integrated into the corresponding compartments of the chip system using a mixture of natural polymers, such as collagen and hyaluronic acid, along with the non-toxic blood coagulating factors fibrinogen and thrombin.

Recent advances in toxicology have highlighted the unique sensitivity of stem cells to toxic substances compared to terminally differentiated cells, such as fibroblasts and endothelial cells 8, 9. *In vitro*, the differentiation potential of stem cells allows them to maintain homeostasis and, when undifferentiated, a heightened responsiveness to toxic exposure, which can significantly impact their viability and function 10, 11. Despite the promising potential of stem cell-based toxicant screening platforms, several limitations must be addressed. A significant challenge is the inherent variability and heterogeneity of stem cell populations 12, 13, which can lead to inconsistent responses and reduced reproducibility of results. Long-term stability and viability maintenance during *in vitro* culture are also significant challenges 14. Prolonged culture can lead to genetic and epigenetic changes that may alter toxicological responses. Immortalized stem cells expressing simian virus 40 (SV40) large T antigen provide a consistent and homogeneous stem cell population 15 with enhanced reproducibility and reliability in toxicological assays. This consistency mitigates the variability seen in primary stem cell cultures, ensuring more uniform responses to toxic substances and improving the robustness of screening results. Tonsil-resident stem cells exhibit robust proliferative capacity and multipotent differentiation potential, enabling them to model a wide range of tissue types and cellular responses 16. Furthermore, these stem cells demonstrate high genomic stability over prolonged culture periods 17, essential for maintaining consistent and reproducible results in drug screening assays.

A critical challenge in developing organ-on-a-chip-based drug screening platforms is the rapid and precise detection of cellular changes in response to substance exposure. Initial changes in gene expression profiles or signaling pathways triggered by toxic exposure are more directly associated with the early stage of toxic events than with the final stage of functional responses, such as cell cycle arrest and apoptosis, which occur at higher thresholds 18. Alterations in gene expression or biomarker levels can occur rapidly with high sensitivity to external stimuli, often preceding observable cellular events. Thus, assessing these initial changes is an effective and accurate approach to rapidly acquiring data related to particular drug candidates' toxicity 19. In this study, we identified plasminogen activator inhibitor-2 (SERPINB2) as a robust biomarker for predicting the onset of oral toxicity, including cell cycle arrest and apoptosis, in human tonsil-resident stem cells. The gene encoding green fluorescent protein (GFP) was incorporated into the regulatory sequence of SERPINB2, facilitating both a straightforward and quantitative assessment of the response of conjugated biomarkers to toxic exposure. This GFP-tagged detection system was then incorporated into the tonsil-resident stem cells embedded within the designated tonsil chamber of the oral-on-a-chip. This study is the first to establish a multi-compartmentalized and multicellular oral system-on-a-chip integrated with the stem cell-based toxicity marker-conjugated GFP detection platform. This system provides precise and rapid toxicity assessments of potential drug candidates. Adverse effects are often undetected by single-cell monolayer platforms and even recent 3D drug screening models.

## Materials and methods

### Isolation and establishment of human oral tissue constituent cells

Human tonsil-derived stem cells were obtained from waste tissue after tonsillectomy with written informed consent from the patients and approval from the Gachon University Institutional Review Board (IRB No: GBIRB2018-133). The tonsil tissue was minced into small pieces that were digested in Dulbecco's modified Eagle's medium (DMEM) containing 10% fetal bovine serum (FBS) and 250 U/ml type I collagenase for 5 h at 37°C in a rotating shaker. The digestion mixture was filtered through a 40-µm cell strainer to separate stromal-like tonsil stem cells from epithelial gland fragments and undigested tissue. The isolated stem cells were cultured in StemPro® MSC SFM CTS™ (Cat No.: A1033201; GIBCO, Franklin Lakes, NJ, USA) at 37°C in an air atmosphere containing 5% CO_2_. The culture medium was changed every two to three days. Human periodontal ligament tissues were meticulously collected from patients undergoing orthodontic treatment at Gachon University Gil Medical Center. Written informed consent was obtained from all the patients. This study was approved by the Institutional Review Board of Gachon University (Institutional Review Board no: GCIRB2019-334). The resected periodontal ligament tissues were mechanically minced into small fragments and enzymatically digested in DMEM containing 10% FBS and 250 U/ml type I collagenase for 5 h at 37°C in a rotating shaker. Following digestion, the solution was filtered through a 70 µm mesh cell strainer to remove the undigested tissue fragments, followed by an additional filtration through a 40 µm mesh cell strainer to isolate the periodontal ligament fibroblasts from the vascular cells and their aggregates. Isolated periodontal ligament fibroblasts were cultured in high-glucose DMEM (Cat. No.: LM001-05; WELGENE Biotech, Taipei, Taiwan) supplemented with 10% FBS and 1% penicillin/streptomycin at 37°C in an atmosphere of 5% CO_2_. Human gingival keratinocytes were obtained from primary gingival keratinocytes (Cat. No.: PCS-200-014; ATCC, Manassas, Virginia, USA) and expanded in high-glucose DMEM (Cat. No.: LM001-05; WELGENE Biotech) and 10% FBS. Human umbilical vein endothelial cells (HUVECs; PCS-100-010 ™, ATCC) were maintained in Endothelial Cell Growth Basal Medium-2 (Lonza, Basel, Switzerland) supplemented with 10% serum and Endothelial Growth Medium-2. The culture medium was refreshed every two days to maintain optimal growth conditions. Normal human cells exhibit limited proliferative capacity *in vitro* 20. The cells were immortalized by stable transfection with the SV40 large T antigen to overcome this limitation, which minimally alters their original characteristics 21. Immortalized cells derived from a single clone provide a more homogeneous population than the heterogeneous nature of primary cultured cells, which often display variable morphologies.

### Construction of oral-on-a-chip platform by injection of PDMS into a 3D-printed casting mold

**Figure 1:**
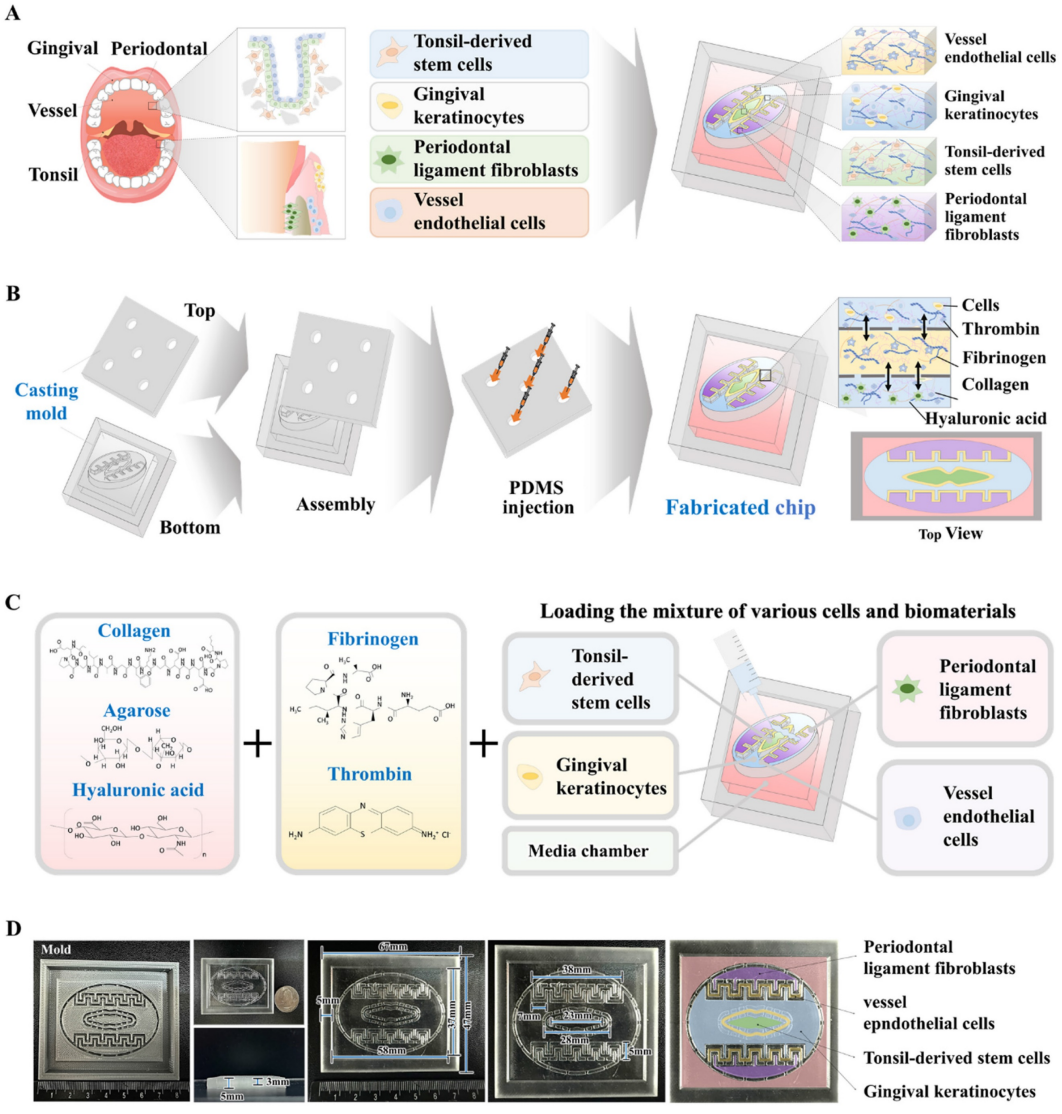
** Fundamental design of the human oral-on-a-chip that effectively replicates the structural and multicellular complexities of oral tissue.** The diagram illustrates the fundamental design of the multi-compartmentalized oral-on-a-chip platform. The dynamic interactions among cells across different compartments include the tonsil stem cell, gingival keratinocyte, and periodontal ligament chambers. This system combines diverse cellular components and 3D structural intricacies made from natural polymers. A thin porous barrier seamlessly connects the adjacent chambers, facilitating bidirectional communication among various embedded cells via the transfer of growth factors and cytokines. Moreover, to effectively replicate the complex multicellular interactions typical of oral tissue environments, these chambers are surrounded by media channels **(A)**. The human oral-on-a-chip system utilizes a 3D casting mold to stably reproduce the structural characteristics of oral tissues, crafted using PLA-based 3D printing. PDMS was subsequently injected into the 3D printed mold, and the resulting chip platform was extracted following polymerization process **(B)**. To accurately mimic the physiological features and multicellular complexity of oral tissue, the PDMS-based oral-on-a-chip platform was embedded with various human oral cell types that included gingival keratinocytes, tonsil-resident stem cells, periodontal ligament fibroblasts, and vascular endothelial cells. The cells were incorporated with a mixture of natural polymers, specifically hyaluronic acid and collagen, and combined with the thrombin and fibrinogen coagulating factors. This tonsil stem cell chamber is supported by adjacent chambers housing human gingival keratinocytes and periodontal ligament fibroblasts, facilitating a comprehensive representation of the oral tissue microenvironment **(C)**. The PDMS-based oral-on-a-chip platform is rectangular (67 × 47 mm, with a height of 8 mm). This device simulates the microenvironment of oral tissues, promoting multicellular interactions between the tonsil stem cell chamber and neighboring compartments containing human gingival keratinocytes periodontal ligament fibroblasts **(D)**.

As shown in Fig. 1B, a casting mold design for the oral-on-a-chip was created using CAD software (Fig. 1B). Upon completion, the 3D CAD data were converted to 3D surface geometric data consisting of various vector types, which were then segmented along the Z-axis with a thickness of 50 μm. The information for each segmented layer was subsequently transferred to a Master EV digital light-processing-based 3D printer (Carima, Seoul, Korea). The casting mold for the oral-on-a-chip device was fabricated from poly(lactic) acid (PLA) using a layer-by-layer manufacturing process.

### Fabrication procedure for the PDMS-based oral-on-a-chip platform

To manufacture the oral-on-a-chip platform, PDMS (Sylgard® 184, Dow Corning Corp., Auburn, MI, USA) was prepared by mixing base and curing agent at a ratio of 20:3. The mixture was thoroughly blended for 10 min at room temperature to ensure homogeneous polymerization. The resulting PDMS solution was injected into a 3D-printed casting mold designed as an oral-on-a-chip. The mold was placed in a vacuum chamber to eliminate dissolved gases. Following degassing, the PDMS-injected mold was cured in an oven at 65°C for 24 h. Once polymerization was complete, the mold was allowed to cool to room temperature. Finally, the fabricated chip was carefully released from the casting mold using a precision razor blade to ensure the chip platform remained intact and undamaged.

### Incorporating diverse types of oral tissue cells in each segment of the constructed chip system

Various oral tissue constituent cells were embedded within the compartments of the fabricated oral-on-a-chip platform. Solutions of type I collagen (3 mg/ml) and hyaluronic acid (3 mg/ml) was prepared in dimethyl sulfoxide (DMSO). A natural polymer mixture containing collagen and hyaluronic acid and blood coagulation factor solutions of 12.5 mg/ml fibrinogen and 1.25 U/ml thrombin was also prepared in DMSO to enhance the mechanical strength. Human tonsil-derived stem cells, periodontal ligament fibroblasts, gingival keratinocytes, and vascular endothelial cells were reconstituted in a 1:1:1:1 mixture of hyaluronic acid (24 mg/ml), collagen (24 mg/ml), fibrinogen (50 mg/ml), and thrombin (5 U/ml). This cell-polymer mixture was loaded into each compartment of the PDMS-based oral-on-a-chip platform using a syringe without additional coating with extracellular matrix (ECM). The mixture was allowed to polymerize by cooling to room temperature over 30 min. An elongated tonsil stem cell chamber 3 mm in diameter and 28 mm thick was formed, with approximately 2 × 10^5^ cells/ml within the tissue construct. The tonsil stem cell chamber was surrounded by a gingival keratinocyte chamber containing human gingival keratinocytes. The chamber containing embedded periodontal ligament cells was positioned at the top and bottom of the chip platform. Consistency was maintained across these three chambers using a uniform ratio of a natural polymer complex comprising type I collagen, hyaluronic acid, and a fibrinogen-thrombin mixture. The culture medium was refreshed every 2 to 3 days to sustain cell viability and function.

### Evaluation of microstructure, compressive strength, and rheological properties of the produced oral tissue constructs within the chip platform

The microstructural characteristics of the natural polymer-based oral tissue architectures were examined using variable pressure and field emission scanning electron microscopy (SEM) using an EVO®LS10 microscope (Carl Zeiss, Jena, Germany). The examination was performed at the Korean Basic Science Institute (Chuncheon, Korea). The fabricated oral tissue samples were freeze-dried and coated with a thin 10 nm layer of gold/palladium for 30 s at a discharge current of 15 mA using an Ion Sputter 1010 device (Hitachi, Tokyo, Japan). Microstructural images were captured at an accelerating voltage of 1.2 to 1.3 kV following established protocols 22.

The compressive stress-strain behavior of the oral tissue architecture was evaluated using a QM100S universal testing machine (QMESYS, Gunpo, Korea). Cylindrical samples of natural polymer-based oral tissue architecture, each with a diameter of 10 mm and a height of 3 mm, were subjected to uniaxial compression tests. A gradual compression force was applied at a displacement rate of 5 mm/min until the samples were fractured, allowing for the calculation of stress at failure in the previously described 22.

Concerning rheological properties, the viscosity of the fabricated tissue architecture was assessed at 37°C using a model MCR 102 rheometer (Anton Paar, Zofingen, Switzerland). The shear rate was varied from 1 to 20 s^-1^ to evaluate potential shear thinning or thickening behavior. This analysis was performed following established protocols to ensure consistency and reliability of the results 22.

### Evaluation of extended cell viability of embedded cells in the oral-on-a-chip

The prolonged viability of cells embedded within the constructed oral-on-a-chip platform was evaluated using a Live & Dead Assay Kit (Cat. No: L3224; Invitrogen Life Technologies, Carlsbad, CA, USA) 1, 7, 14, 21, and 28 days following embedding, according to the manufacturer's instructions. Each chip compartment was rinsed three times with serum-free DMEM to prepare for the assay. One milliliter of the assay solution containing 2 mM ethidium homodimer-1 and 4 mM calcein AM was introduced into each chamber. Following a 30-minute incubation at room temperature, the compartments were examined using the EVOS FL Cell Imaging System (Thermo Fisher Scientific, Waltham, MA, USA) to assess cell viability.

### Assessment of prolonged metabolic activities of embedded cells in the oral-on-a-chip

The extended metabolic activities of the cells embedded within the oral-on-a-chip were evaluated using the CCK-8 assay (Cat. No. KTC011001; Abbkine, Atlanta, GA, USA) according to the manufacturer's instructions. Each compartment received 100 µl/ml of CCK-8 solution in serum-free DMEM. The chip containing the cells was then incubated for 4 h at 37°C in a 5% CO_2_ incubator. Metabolic activity was quantified by measuring absorbance at 450 nm using a SoftMax Pro 5 microplate reader (Molecular Devices, San Jose, CA, USA).

### Antibody-based fluorescence staining of embedded cells in the oral-on-a-chip

For fluorescence staining, samples were fixed with 4% paraformaldehyde and then permeabilized using a solution containing 0.4 M glycine and 0.3% Triton X-100. To prevent nonspecific binding, the samples were blocked with 2% normal swine serum (DAKO, Glostrup, Denmark). Staining was performed as previously described 23 using primary antibodies to GFP (Cat. No: V820-20; Invitrogen), platelet endothelial cell adhesion molecule (PECAM1, Cat. no. BBA7; R&D Systems, Minneapolis, MN, USA), Von Willebrand factor (vWF, Cat. No.: ab6994; Abcam, Cambridge, UK), vimentin (Cat. No.: 550513; BD Biosciences, Santa Clara, CA, USA), fibronectin (Cat. No.: ab2413; Abcam), cytokeratin 5 (Cat. No.: ab52635; Abcam), cytokeratin 8 (Cat. No.: sc-8020; Santa Cruz Biotechnology, Dallas, TX, USA), CD105 (Cat. No.: 130-094-941; Miltenyi Biotec, Bergisch Gladbach, Germany), and NANOG (Cat. No.: sc-374001; Santa Cruz Biotechnology). The expression patterns of these proteins were visualized and analyzed by fluorescence microscopy using a model LSM 510 Meta instrument (Carl Zeiss).

### Real-time PCR analysis

Total RNA was extracted using TRIzol reagent (Invitrogen Life Technologies) following the manufacturer's protocol. The purity of isolated RNA was confirmed by assessing the 260/280 nm absorbance ratio. First-strand cDNA synthesis was performed using 1 μg of total RNA using SuperScript II (Invitrogen Life Technologies). Ten percent of the synthesized cDNA was used in each PCR mixture, which included the Express SYBR Green qPCR Supermix (BioPrince, Seoul, South Korea). Real-time PCR was performed using the Rotor-Gene Q Real-Time PCR Cycler (QIAGEN, Valencia, CA, USA). PCR involved 40 cycles of denaturation at 95°C for 20 s, annealing at 60°C for 20 s, and extension at 72°C for 25 s. The relative mRNA expression levels of the target genes were normalized to the expression of peptidyl-prolyl isomerase A and calculated using the ΔΔCT method. The primer sequences used for the PCR are listed in Table 1.

### Protein extraction and immunoblotting

Cells were lysed in a buffer composed of 50 mM Tris, 5 mM EDTA, 150 mM NaCl, 1 mM dithiothreitol, 0.01% NP-40, and 0.2 mM phenylmethylsulfonyl fluoride. The protein concentrations in the lysates were determined using bovine serum albumin solutions of known concentrations as standards. Equal amounts of protein from each sample were separated based on their molecular weights using sodium dodecyl sulfate-polyacrylamide gel electrophoresis. Following electrophoresis, the proteins were electrophoretically transferred onto nitrocellulose membranes (Bio-Rad, Hercules, CA, USA). The membranes were blocked with 5% (w/v) nonfat dry milk in Tris-buffered saline containing Tween-20 (TBS-T) for 1 h at room temperature. Subsequently, the membranes were incubated overnight at 4°C with primary antibodies to SERPINB2 (Cat. No: ab47742; Abcam), matrix metalloproteinase 2 (MMP-2, Cat. #4022; Cell Signaling Technology, Danvers, MA, USA), MMP-9 (Cat. #13667; Cell Signaling Technology), caspase-3 (Cat No. #9662; Cell Signaling Technology), and β-actin (Cat No.: ab189073; Abcam). After incubation with the primary antibody, the membranes were washed and incubated with horseradish peroxidase-conjugated secondary antibodies, including goat anti-rabbit IgG (554021; BD Pharmingen, San Diego, CA, USA) and goat anti-mouse IgG (554002; BD Pharmingen), for 60 min at room temperature. Bound antibodies were visualized using an enhanced chemiluminescence reagent.

### Transwell invasion/migration experiment

To evaluate cell migration, cells (1 × 10^5^ per well) were placed in the upper chambers of a Transwell plate (Corning Inc., Corning, NY, USA), which featured 8.0 μm pores within 6.5 mm-diameter polycarbonate membranes. The Transwell system was arranged in a 24-well plate. Nonmigrating cells remaining on the upper surface of the membrane were removed by gentle scrubbing with laboratory paper. Cells that successfully migrated to the lower side of the membrane were fixed with 4% paraformaldehyde for 5 min and stained with hematoxylin for 15 min. The number of migrating and invading cells was counted in three randomly selected fields per well using an optical microscope at 50× magnification.

### Targeted suppression of SERPINB2 via specific short hairpin RNAs (shRNAs)

To achieve targeted SERPINB2 knockdown, specific shRNA (accession no. NM_002575) and scrambled control shRNA were obtained from Bioneer (Daejeon, South Korea). According to the manufacturer's protocol, transfections were performed using Lipofectamine 2000 (Cat. No. 52887; Invitrogen). The shRNA targeting SERPINB2 (3 μg/ml) was combined with 3 μl of Lipofectamine 2000 in serum- and antibiotic-free Opti-MEM (Gibco). Five hours before transfection, the Opti-MEM was replaced with fresh StemPro MSC SFM CTS medium (Thermo Fisher Scientific) containing 10% serum. The SERPINB2 shRNA construct, optimized to achieve high transfection efficiency at the mRNA level, was used for gene silencing.

### Examination of Gene Expression Omnibus (GEO) datasets

GEO (https://www.ncbi.nlm.nih.gov/geo/) is an extensive international repository for wide-ranging gene expression datasets obtained from RNA-seq, DNA microarrays, and chip sequencing 24, 25. The GEO datasets were systematically organized into four primary sections: experimental designs, raw data, groups, and platforms. The results from each dataset were further classified based on several factors, including treatment conditions, physiological conditions, and disease states. These categorized data were displayed as "GEO profiles" featuring rank measurements, functional annotations, and charts showing expression values for each gene across all analyzed samples 26. Data were analyzed according to established protocols to assess the expression profile of SERPINB2 in response to exposure to various toxic materials 26.

### Statistical analysis

Statistical analysis was performed using GraphPad Prism 9.0 (GraphPad Software, San Diego, CA, USA). Two-tailed Student's t-test was used to evaluate the data. Statistical significance was set at *p* < 0.05.

## Results

### Development of an intricately compartmentalized oral-on-a-chip device that properly replicates the complex structure and cellular diversity of oral tissue

Fig. 1A depicts the core concept of the multi-segmented oral-on-a-chip device. The device facilitates dynamic multicellular crosstalk among the tonsil, periodontal ligament, and gingival keratinocyte chambers, using a design that integrates diverse cellular elements and 3D structural complexities made of natural polymers. A thin porous separator directionally interconnects these neighboring chambers, facilitating bidirectional interactions among the chambers by allowing the transfer of diverse regulatory peptides and growth factors. Fabrication of the oral-on-a-chip system was accomplished using a series of sequential phases. To uniformly and reliably fabricate the system that maintains the intricate compartmental architecture of oral tissue, the initial step involved crafting a casting mold for the chip structure using 3D printing technology with PLA. PLA was selected because of its superior printing capabilities, which enable the creation of detailed and intricate designs, and its excellent mechanical stability, which ensures its structural reliability and durability 27. Subsequently, PDMS was injected into the 3D-printed mold and solidified at room temperature. Next, the multichambered oral-on-a-chip device was carefully extracted from the casting mold (Fig. 1B). In the constructed rectangular PDMS-based oral-on-a-chip device (67 mm × 47 × 8 mm^3^), a centrally located tonsil chamber is integrated with human tonsil-resident stem cells. The diameter of 23 mm closely mimics the anatomical structure of the tonsils within the oral cavity. The tonsil chamber is surrounded by a chamber containing human gingival keratinocytes, which support the chamber by exchanging various growth factors and cytokines. A chamber containing embedded human periodontal ligament cells is positioned at the top and bottom of the chip platform (Fig. 1C). In the use of the chip system to replicate the diverse cellular architecture of the oral tissue, a variety of oral tissue cells, including gingival keratinocytes, tonsil-resident stem cells, periodontal ligament fibroblasts, and vascular endothelial cells, were combined with a biocompatible matrix composed of collagen and hyaluronic acid. Additionally, to reinforce the physical properties of the cell-embedded natural polymer matrix, non-toxic blood coagulating factors, specifically fibrinogen and thrombin, were employed as crosslinking agents. This mixture of diverse oral cells and polymer matrix was then incorporated into the designated chambers of the segmented oral-on-a-chip platform (Fig. 1D).

### Diverse physical properties of the natural polymer-derived tissue matrix within the oral-on-a chip device: microscopic architecture, structural integrity, and rheological properties

**Figure 2:**
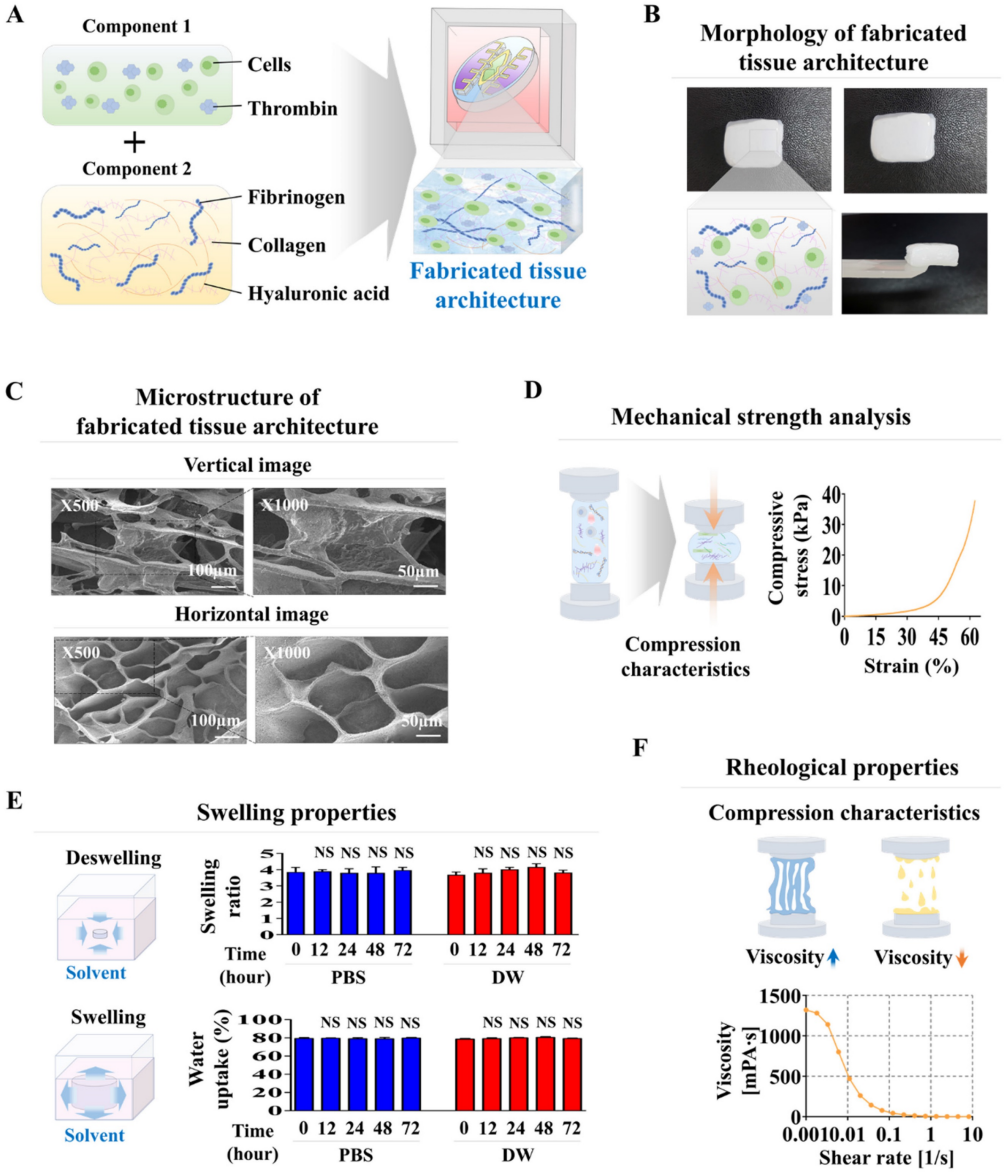
** Development of oral tissue model using natural polymers and analysis of structural characteristics.** The various cell types found in oral tissue were combined with a mixture of natural polymers, such as hyaluronic acid and collagen, and supplemented with the non-toxic coagulants thrombin and fibrinogen **(A)**. The fabricated tissue architecture has a refined surface, soft texture, and is white in color. **(B)** Cross-sectional and lateral views of the tissue matrix were analyzed using scanning electron microscopy (SEM) to explore their microstructural characteristics. SEM images revealed a uniformly distributed porous network within the tissue constructs, characterized by pores with diameters of 50 to 100 μm. This structure emerged from the interaction of natural polymers with blood coagulation factors **(C)**. The mechanical properties of the fabricated tissue structures were assessed using a universal testing machine, which applied single-axis compression forces. Samples of the tissue, each 10 mm in diameter and height, were exposed to different levels of uniaxial compressive stress to analyze their mechanical response. To assess the exact breaking point of the fabricated samples, a uniaxial compression test was performed with a consistent load rate of 5 mm/min until failure occurred in the samples **(D)**. To assess the absorption properties of the tissue models, the fabricated samples were submerged in distilled water and PBS (pH 7.4) at 37°C for 24 h. Following hydration, excess fluid was removed, and the samples were weighed to measure their water absorption capability **(E)**. Viscosity measurements of the fabricated tissue structures were performed over a shear rate spectrum from 1 to 10/s. Viscosity markedly declined from about 1000 to 0 Pa-sec as the shear rate increased **(F)**. All experiments were performed in triplicate. Significant differences are indicated as follows: *, *p* < 0.05; **, *p* < 0.005; and ***, *p* < 0.001 (two-sample t-test).

Collagen combined with hyaluronic acid is frequently employed in numerous tissue engineering endeavors to mimic the tissue microenvironment, owing to its reliable structural integrity, uniform consistency, and ideal biocompatibility 28-30. These natural polymer-derived tissue matrices create a supportive framework that enhances cell attachment and survival, while facilitating crucial signaling pathways that govern various cellular functions, such as self-renewal, movement, and differentiation 31, 32. However, the mechanical strength of these natural polymer-derived tissue matrices is inadequate to accurately replicate the physical properties of diverse human tissues. Consequently, to improve their structural integrity while avoiding cytotoxic effects, the non-toxic blood coagulating compounds fibrinogen and thrombin were utilized as natural crosslinkers (Fig. 2A). The tissue matrix fabricated from various combinations of thrombin and fibrinogen, and the natural polymers hyaluronic acid and collagen within each segment of the chip platform had a consistent surface and soft white texture (Fig. 2B). The microarchitecture of the developed tissue matrix in each segment of the chip was examined by SEM. The findings revealed a homogeneously dispersed porous structure across the tissue matrix with pore sizes varying between approximately 50 to 100 μm (Fig. 2C). The highly porous structure of these biomaterial-based 3D tissues promotes efficient exchange of nutrients, oxygen, and waste products, ensuring the maintenance of cellular viability and functionality.

Its complex microporous architecture is formed by the consistent bonding of hyaluronic acid and collagen fibers, which blood-coagulating agents crosslink. This porous and non-cytotoxic structure improves cellular adhesion and increases survival rate 33. Moreover, a substantial challenge of natural polymer-derived tissue matrices is sustaining adequate mechanical strength to preserve their structural integrity, essential for supporting the adhesion and survival of embedded cells 34, 35. The structural robustness of the presently fabricated natural polymer-derived tissue matrix was approximately 40 kPa (Fig. 2D). Assessment of the swelling properties of fabricated tissue matrix, performed in both PBS and distilled water at pH 7.4 and 37°C, indicated that both structural integrity and volume were predominantly maintained after absorbing water (Fig. 2E). Rheological behavior, particularly viscosity, is essential for the functionality of biomaterial-derived tissue matrices because it significantly influences the viability of the embedded cells 36. For instance, a natural polymer-derived tissue matrix with low viscosity can successfully preserve its structural integrity; however, this often leads to decreased survival of the cells within it 37. The viscosity of the created tissue matrix was assessed by analyzing the dynamic viscosity at different shearing speeds. As the shearing speed increased from 1 to 10/s, the rheological behavior of the created tissue matrix gradually weakened, decreasing from approximately 1300 to 0 Pa s (Fig. 2F). These findings demonstrate the suitable physical characteristics of the natural polymer-derived tissue matrix for embedding diverse cells derived from oral tissues.

### Assessment of biocompatibility for diverse oral tissue cells embedded within the oral-on-a-chip device: cell distribution, long-term viability, metabolic activity, and molecular characteristics

Although natural polymer-derived tissue matrices exhibit ideal physical characteristics for cell encapsulation, several technical challenges remain unaddressed. These include achieving uniform cell distribution and sustained embedded cell survival, maintaining their intrinsic cellular properties, and ensuring functional integrity within the tissue matrix. Consequently, we analyzed the distribution patterns, long-term survival rates, sustained metabolic activities, and molecular characteristics of diverse oral cells embedded within a natural polymer-derived tissue matrix. Four distinct oral tissue cell types were integrated into the respective compartments of the chip system. Initially, human tonsil-resident stem cells were freshly isolated from the healthy tonsil tissue of patients undergoing tonsillectomy and expanded *in vitro* (Supplementary Fig. 1A). The cells were examined by flow cytometry to verify stem cell characteristics by measuring the expression of several stemness/pluripotency markers, including CD34, CD44, CD45, CD73, and CD105 (Supplementary Fig. 1B). In addition, their capacity for multilineage differentiation was evaluated by examining their ability to differentiate into adipocytes and osteoblasts (Supplementary Fig. 1C). Real-time PCR was used to analyze the expression patterns of pluripotency-associated genes, which serve as indicators of stem cell differentiation potential (Supplementary Fig. 1D). Ligament fibroblasts freshly isolated from healthy human periodontal tissue were morphologically characterized by their polygonal shape (Supplementary Fig. 2A) and notably high levels of specific cellular markers, including fibronectin and vimentin (Supplementary Fig. 2B). HUVECs have been utilized as representative models of vascular cells, given their extensive use in diverse vascular studies 38. These cells were characterized by their circular shape (Supplementary Fig. 3A). They exhibited positive immunostaining for PECAM-1 and vWF (Supplementary Fig. 3B). Gingival keratinocytes were characterized by their polygonal morphology and significant self-renewal capacity (Supplementary Fig. 4A), and displayed pronounced expression of the established biomarkers, cytokeratin 5 and 8 (Supplementary Fig. 4B). The integration of blood vessel endothelial cells will allow us to model the vascular microenvironment of the oral cavity, which plays a crucial role in nutrient delivery, waste removal, and cellular responses to toxicants. Gingival keratinocytes and periodontal ligament fibroblasts are also key components of the oral cavity's structural integrity and are directly involved in the maintenance of oral tissues and their response to injury and toxins.

**Figure 3:**
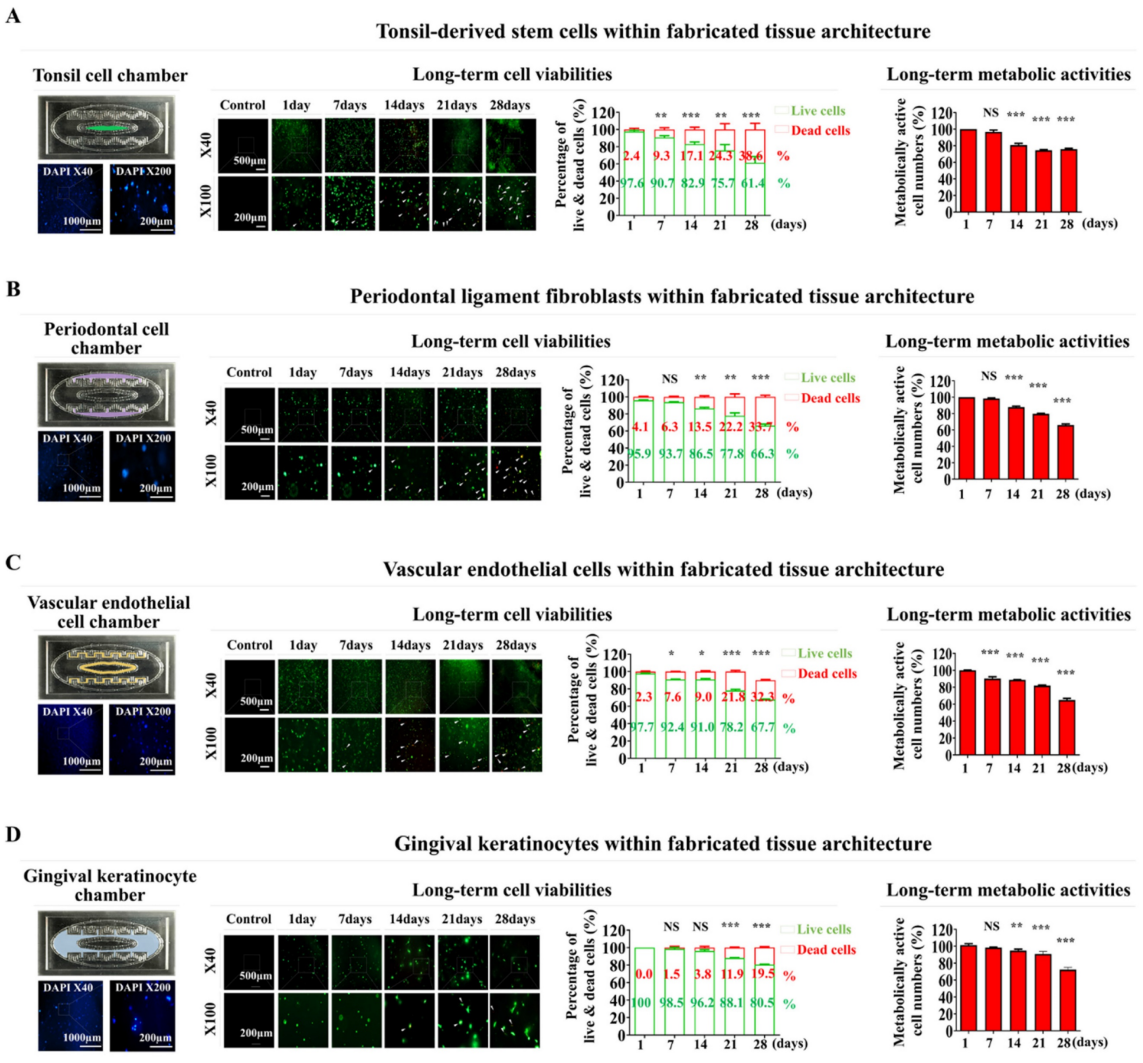
** Analysis of sustained viability and metabolic functions of multiple cells embedded within each segment of the oral-on-a-chip model.** The arrangement of various cell types within each segment of the chip was assessed. This involved culturing the tissue constructs in a cell-specific medium for 24 h, followed by staining with the DNA-specific dye 4′,6-diamidino-2-phenylindole (DAPI) to visualize the cells' spatial distribution. Fluorescent microscopy was utilized to examine the distribution patterns of cells throughout the tissue structures on the chip. The oral tissue cells in the chip compartments were tonsil-derived stem cells** (A)**, periodontal ligament fibroblasts **(B)**, vascular endothelial cells **(C)**, and gingival keratinocytes **(D)**. Subsequently, cells were placed in medium specialized for each cell type, and cultured for 1, 7, 14, 21, or 28 days after integration. Viability was evaluated with a commercial assay using fluorescent dyes to differentiate live (green) and dead (red) cells. The extended viability of the cells in each chamber was determined by fluorescent imaging. Over 60% of the cells embedded within each segment of the chip remained viable for 28 days. Furthermore, each segment was exposed to a serum-free environment with CCK-8 solution for 48 h. The metabolic activity of these cells was measured by assessing the optical density at 450 nm. All experiments were performed in triplicate. Significant differences are indicated as: *, *p* < 0.05; **, *p* < 0.005; and ***, *p* < 0.001 (two-sample t-test).

Subsequently, these established oral tissue cells were integrated into a natural polymer-based tissue matrix comprised of hyaluronic acid and collagen and combined with the thrombin and fibrinogen blood-coagulating proteins. Subsequently, they were integrated into each segment of the oral cavity-on-a-chip device. Nuclear staining was performed to assess the spatial arrangement of fabricated chip devices. Maintaining a uniform distribution of cells throughout the chip device is essential for maintaining organ-specific activity and ensuring reliable, consistent, and accurate experimental results 33. In the novel chip developed in this study, embedded cells were equally scattered throughout the natural polymer-derived tissue matrix within the chip (Fig. 3A-D). Viability assessments were performed at several time points to determine the extended survival of integrated cells within the oral cavity of the chip device. Although there was a steady decrease in cell survival rates, over 83% of the embedded cells remained viable after 2 weeks, and approximately 60% continued to survive after 4 weeks (Fig. 3A-D). The extended metabolic activities of cells incorporated orally on the chip were also assessed weekly using the CCK-8 assay after cell integration. Although the prolonged metabolic activities of numerous engrafted cells decreased gradually, approximately 70% of the cells within the chip device maintained their metabolic activities after 28 days (Fig. 3A-D).

**Figure 4:**
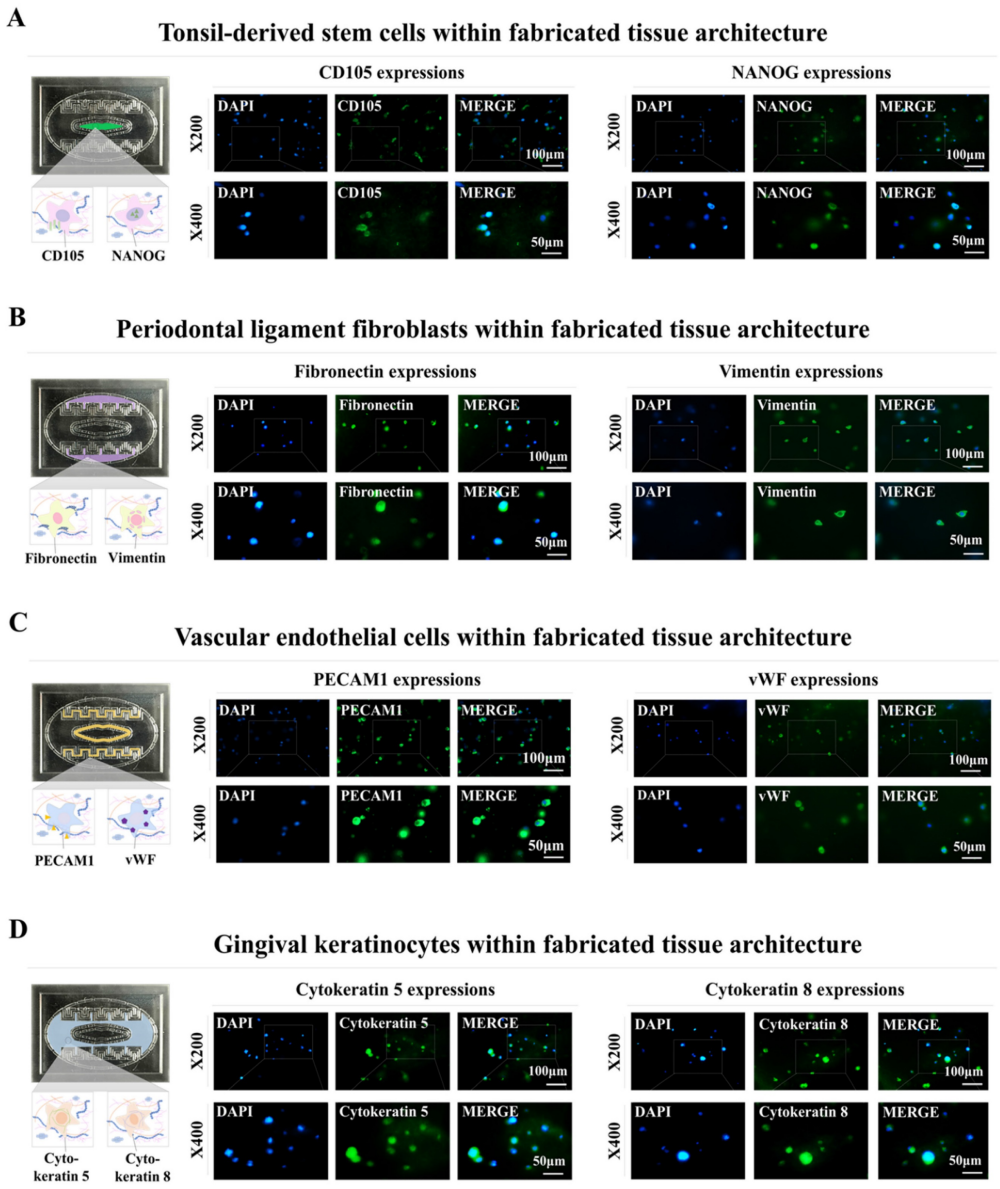
** Preservation of distinct molecular properties within the natural polymer-based tissue matrix.** Evaluations were conducted to verify whether various cell types maintained their molecular characteristics after being incorporated into specific tissue microenvironment of natural polymer-based tissue matrix. This process involved culturing the cells in specially formulated media for one week, followed by their evaluation using biomarkers unique to each cell type. Tonsil-derived stem cells were stained to identify CD105 and NANOG **(A)**, while periodontal ligament fibroblasts were assessed for fibronectin and vimentin **(B)**. Vascular endothelial cells were marked with labels for PECAM1 and vWF **(C)**, and gingival keratinocytes were evaluated using markers for cytokeratin 5 and 8 **(D)**. Each experiment was conducted three times. The nuclei in each field were stained with DAPI.

Distinct biomarkers were used to examine the integrated cells within the fabricated oral-on-a-chip device to determine whether they retained their inherent molecular profiles. The CD105 and NANOG molecular markers of tonsil-resident stem cells were prominently expressed in the integrated cells (Fig. 4A). The fibronectin and vimentin cytoskeletal components, which are vital for fibroblast migration and invasion, were predominantly expressed in the periodontal ligament fibroblasts (Fig. 4B). Within the chip device, the embedded vascular endothelial cells showed stable expression of PECAM1 and vWF, which are established markers for this cell type (Fig. 4C). Embedded human gingival keratinocytes also revealed significant levels of the cytokeratin 5 and 8 markers (Fig. 4D). These results indicate that different types of embedded cells maintain their inherent molecular profiles within the biomaterial-derived tissue matrix of the chip device over extended culture periods.

### Identification of SERPINB2 stable indicator for estimating oral toxicity in human tonsil-resident stem cells

**Figure 5:**
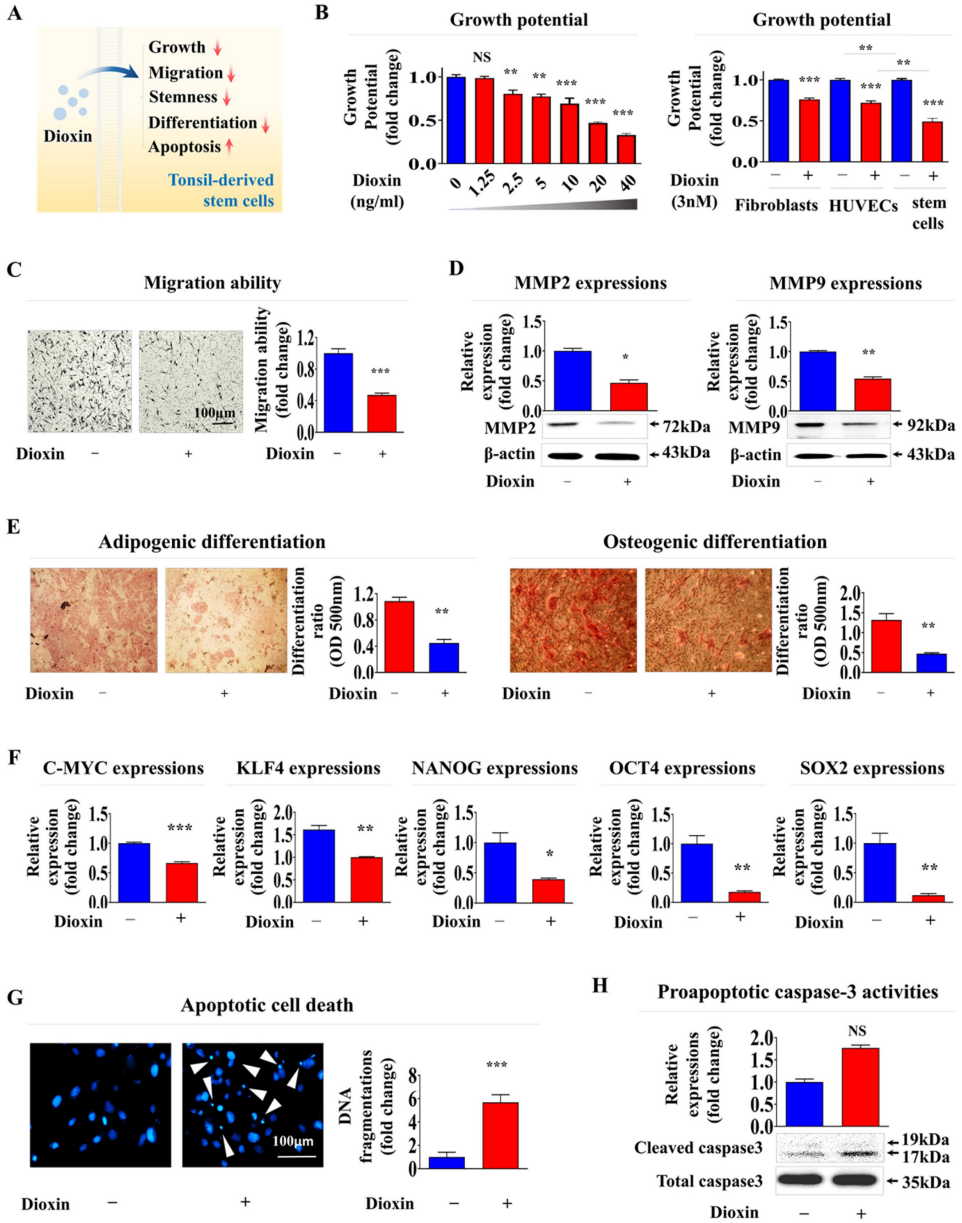
** Treatment with the reference toxicant dioxin significantly inhibits various tonsil-derived stem cell functions *in vitro*.** The effectiveness of dioxin was confirmed by its ability to trigger toxicity in tonsil-derived stem cells, measured at specific concentrations and durations of exposure **(A)**. The effect of dioxin on tonsil-derived stem cell renewal was studied by administering various doses (1.25, 2.5, 5, 10, 20, and 40 ng/ml) and measuring the impact after 72 h using the (3-(4, 5-dimethylthiazolyl-2)-2, 5-diphenyltetrazolium bromide) (MTT assay. The impact of dioxin on cell survival was also assessed over 72 h using the same an MTT and fibroblasts, and vascular endothelial cells. Cell viability was quantified as a percentage compared to cells treated with a vehicle control **(B)**. Tonsil-derived stem cells exposed to dioxin (5 ng/ml for 72 h exhibited reduced migratory abilities in a Transwell migration assay, compared to control cells **(C)**. The effect of dioxin on the expression of the key cell migration regulators MMP-2 and MMP-9 was quantified using western blot analysis **(D)**. Tonsil-derived stem cells were cultivated in medium designed to promote adipocyte or osteoblast differentiation; the cells were untreated or exposed to dioxin. The adverse effects of dioxin on the differentiation of the stem cells into adipocytes and osteoblasts were assessed using Oil Red O and Alizarin Red staining techniques. Quantitative analyses of calcium accumulation and lipid formation in the differentiated cells was performed by measuring the absorbance at 500 nm and 570 nm, respectively **(E)**. The impact of exposure to dioxin on the expression of the pluripotency marker genes C-MYC, KLF4, NANOG, OCT4, and SOX2 in cells was assessed using real-time PCR **(F)**. After a 72-h incubation with dioxin, the induced apoptotic DNA condensation and fragmentation were examined using DAPI staining **(G)**. Concurrently, tonsil-derived stem cells that were untreated or cultured with dioxin were analyzed for increased levels of cleaved caspase-3 via western blotting to assess the apoptotic response to dioxin exposure **(H)**. DAPI was used to label nuclei. β-Actin was used as the internal control. All experiments were performed in triplicate. The data are presented as the mean ± standard deviation. *, *p* < 0.05; **, *p* < 0.005; and ***, *p* < 0.001 (two-sample t-test).

A reference toxicant must first be selected to develop a stem cell-based toxic substance screening platform integrated with biomarker analysis. In the present study, the reference toxicant was dioxin. It was selected based on its high ranking in hazardous material classifications by five international authoritative bodies: the Association Advancing Occupational and Environmental Health, the European Chemicals Agency, the International Agency for Research on Cancer, the National Toxicology Program, and the United States Environmental Protection Agency 9. Subsequently, we validated the ability of dioxin to induce toxicity in stem cells at defined concentrations and exposure durations (Fig. 5A). The self-renewal capacity of tonsil-resident stem cells decreased in a concentration-dependent manner upon treatment with dioxin (Fig. 5B). Notably, dioxin exposure had a significantly more significant impact on stem cell function than on fully differentiated somatic cells, such as dermal fibroblasts and vascular endothelial cells, suggesting that undifferentiated stem cells tend to exhibit greater susceptibility to toxic substances than terminally differentiated cells (Fig. 5B). Toxic exposure substantially reduced the migration potential of tonsil-resident stem cells (Fig. 5C). Consistently, Lee et la. also found that toxic exposure more profoundly affected the growth of human umbilical cord blood (UCB)- derived stem cells than terminally differentiated dermal fibroblasts 9. To further confirm the inhibitory effect on migratory ability, western blot analysis was performed to measure the expression levels of MMP-2 and -9, which are crucial for facilitating cell migration/invasion by breaking down the ECM (Fig. 5D). Additionally, toxicity markedly reduced the capacity of tonsil stem cells to differentiate into adipocytes and osteoblasts (Fig. 5E). Similarly, the mRNA levels of the pluripotency-related factors C-MYC, Krüppel-like factor 4 (KLF4), NANOG, Octamer-binding transcription factor 4 (OCT4), and Sex-determining region Y-box 2 (SOX2) in tonsil stem cells were substantially reduced by toxic exposure (Fig. 5F). Toxic exposure also led to increased apoptotic DNA fragmentation (Fig. 5G) and elevated proapoptotic caspase-3 activity (Fig. 5H).

**Figure 6:**
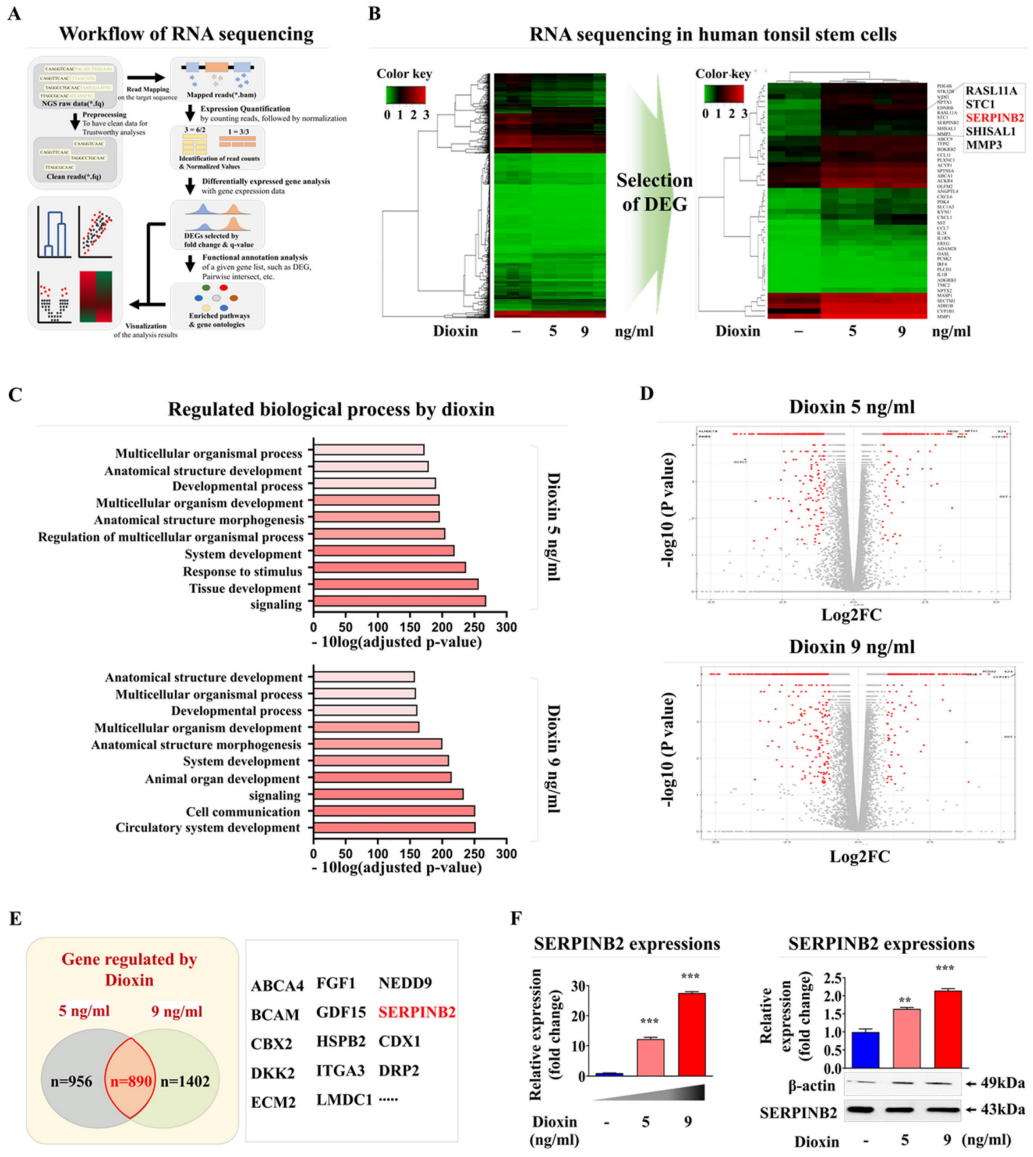
** Identification and validation of a reliable marker predicting oral toxicity using human tonsil-derived stem cells.** Illustration of the fundamental steps in the RNA-seq process, including the experimental setup, alignment of sequences, quantification, and visual depiction of data **(A)**.The RNA-seq data was visualized in a heatmap, showing gene expression differences between control groups and those treated with low (5 ng/ml) and high (7 ng/ml) doses of athe reference toxicant, dioxin. This visualization displays upregulated genes in red and downregulated genes in green, compared to mRNA levels in the control samples **(B)**. KEGG pathway analysis performed to explore the interrelated pathways and functions affected by exposure to dioxin** (C)**. In the array of genes with altered expression, SERPINB2 expression in human tonsil-derived stem cells was significantly positively correlated with the response to dioxin concentrations of 5 ng/ml **(D)** and 9 ng/ml **(E)** dioxin exposure**.** Real-time PCR and western blot verification of the upregulation of SERPINB2 levels in response 5 and 9 ng/ml doses of dioxin **(F)**. β-Actin was used as the internal protein control, and *peptidyl-prolyl cis-trans isomerase* (*PPIA*) was used as the housekeeping gene for real-time PCR. All experiments were performed in triplicate. The data are presented as the mean ± standard deviation. *, *p* < 0.05; **, *p* < 0.005; and ***, *p* < 0.001 (two-sample t-test).

Based on these results, we aimed to develop a stem cell-based screening platform using a biomarker-conjugated detection system to measure oral toxicity. Accordingly, a reliable detection marker was identified through a comprehensive analysis of broad-spectrum genetic transcription profiles by RNA-seq in human tonsil-resident stem cells exposed to dioxin. Fig. 6A provides a detailed schematic summary of the multiple stages of RNA-seq analysis performed on human tonsil-resident stem cells. The expression of many genes was significantly upregulated in human tonsil-resident stem cells following exposure to dioxin concentrations of 5 and 9 ng/ml (Fig. 6B). Kyoyo Encyclopedia of Genes and genomes (KEGG) pathway analysis was performed to evaluate the molecular interactions between diverse signaling pathways and toxic exposure. Exposure to the dioxin reference toxicant notably diminished the activity within the signaling networks crucial for self-renewal and survival, specifically affecting receptor-mediated signaling activity and cytokine receptor affinity (Fig. 6C). In stem, cells exposed to both 5 and 9 ng/ml dioxin, an apparent positive correlation was observed between increased expression of SERPINB2 and toxic exposure in human tonsil-resident stem cells (Fig. 6D and 6E). The elevation in SERPINB2 expression following exposure to dioxin was verified by qPCR and western blotting (Fig. 6F). To confirm the responsiveness of SERPINB2 to a broader range of toxic substances, we have expanded our study by investigating the responsiveness of SERPINB2 to various toxic agents beyond dioxin. The GEO dataset was used to verify further the relationship between elevated SERPINB2 expression and exposure to various toxic substances, including benzo [a]pyrenediol epoxide, nickel chloride, lethal irradiation, ultrafine particles, and oxidized lipids (Supplementary Fig. 5). In addition, our findings also revealed an apparent positive correlation between increased SERPINB2 expression in human tonsil-resident stem cells and exposure to a range of recognized toxicants (Supplementary Fig. 6). These results provide clear evidence confirming that SERPINB2 serves as a universal marker for oral toxicity.

### Verification of the SERPINB2 biomarker by specifically depleting its expression using shRNA

**Figure 7:**
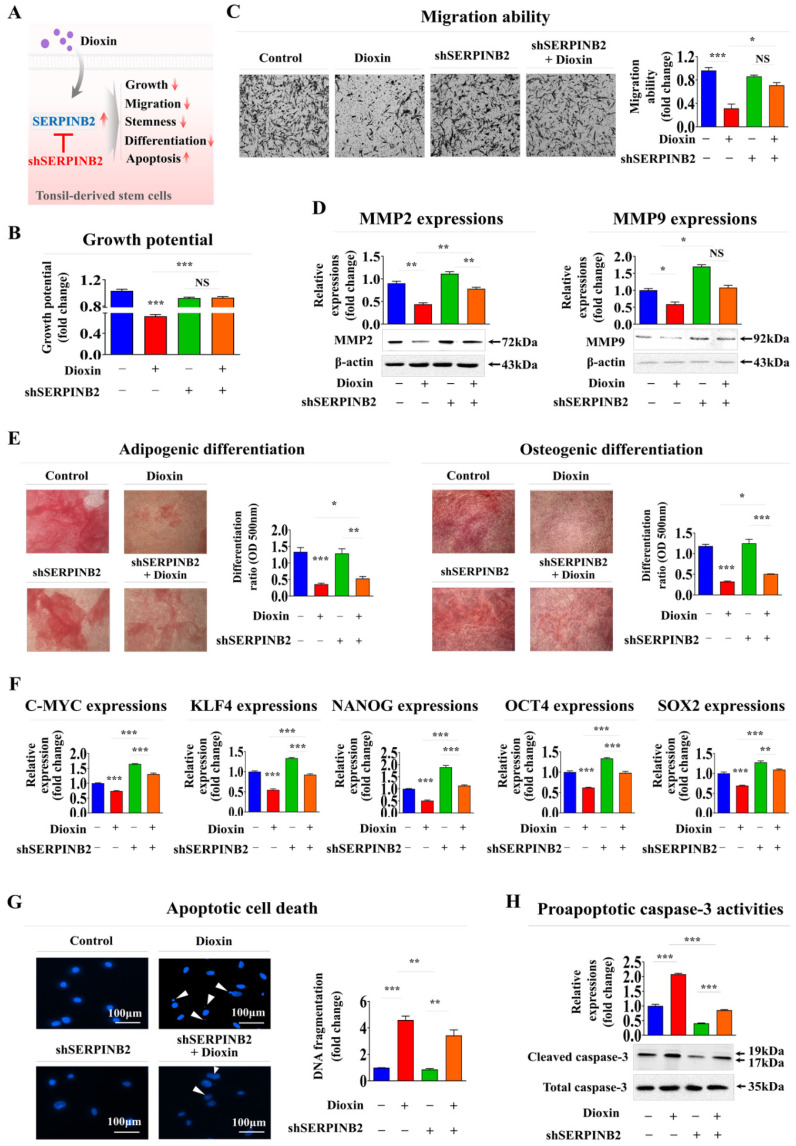
** Validating the reliability of SERPINB2 as a marker for oral toxicity in human tonsil-derived stem cells**. Schematic illustration outlining the role of SERPINB2 in mediating the adverse effects triggered by dioxin in human tonsil-derived stem cells **(A)**. Stem cells transfected with SERPINB2-specific shRNA were either untreated or exposed to dioxin (5 ng/ml) for 72 h. The effects on growth were analyzed using an MTT assay to assess the attenuating effects of SERPINB2 knockdown **(B)**. Depleting SERPINB2 expression effectively counteracts the adverse effects of dioxin on the mobility of tonsil-derived stem cells, as validated by the Transwell migration/invasion assay **(C)** and western blot analysis of the production of MMP-2 and MMP-9 proteins **(D)**. Human tonsil-derived stem cells were transfected with shRNA targeting SERPINB2 and exposed to 5 ng/ml dioxin. The cells were assessed for their differentiation into adipocytes and osteoblasts using Oil Red O and Alizarin Red staining **(E)**. Changes in the gene expression of pluripotency markers C-MYC, KLF4, NANOG, OCT4, and SOX2 in cells assessed using real-time PCR **(F)**. DNA fragmentation linked to apoptosis and caspase-3 related apoptotic processes were evaluated using nuclear staining techniques **(G)** and western blot analysis **(H)**, respectively. β-actin was used as an internal control. All experiments were performed in triplicate. Data are presented as mean ± standard deviation. *, *p* < 0.05; **, *p* < 0.005; and ***, *p* < 0.001 (two-sample t-test).

To determine whether SERPINB2 acts as a critical mediator of multiple cellular activities related to oral toxicity, its expression was suppressed in human tonsil-resident stem cells using a specific shRNA targeting SERPINB2 (Supplementary Fig. 7). Subsequent analyses focused on the effects of SERPINB2 suppression on various cellular processes, such as apoptosis, self-renewal, and migratory capacity, with and without toxic exposure (Fig. 7A).

Notably, SERPINB2 depletion in human tonsil-resident stem cells substantially countered the suppression of cell growth observed in the presence of dioxin (Fig. 7B). SERPINB2 knockdown in human tonsil-resident stem cells significantly reversed the toxic exposure-induced reduction in cellular motility (Fig. 7C) and the levels of MMP-2 and MMP-9 (Fig. 7D). Furthermore, SERPINB2 knockdown significantly alleviated the detrimental effects of toxic exposure on the ability of tonsil-resident stem cells to trans-differentiate into adipocytes and osteoblasts (Fig. 7F). Likewise, the toxic exposure-mediated inhibitory effects on the expression of the pluripotency-associated genes C-MYC, KLF4, NANOG, OCT4, and SOX2 in tonsil stem cells were markedly attenuated by SERPINB2 depletion (Fig. 7E). SERPINB2 knockdown substantially reduced the activation of apoptosis-related DNA breakage (Fig. 7G) and levels of apoptotic cleaved caspase-3 (Fig. 7H) triggered by toxic exposure. These results indicate that SERPINB2 acts as a critical mediator of multiple cellular activities related to oral toxicity.

### Development of GFP-conjugated platform for detecting oral toxicity

**Figure 8:**
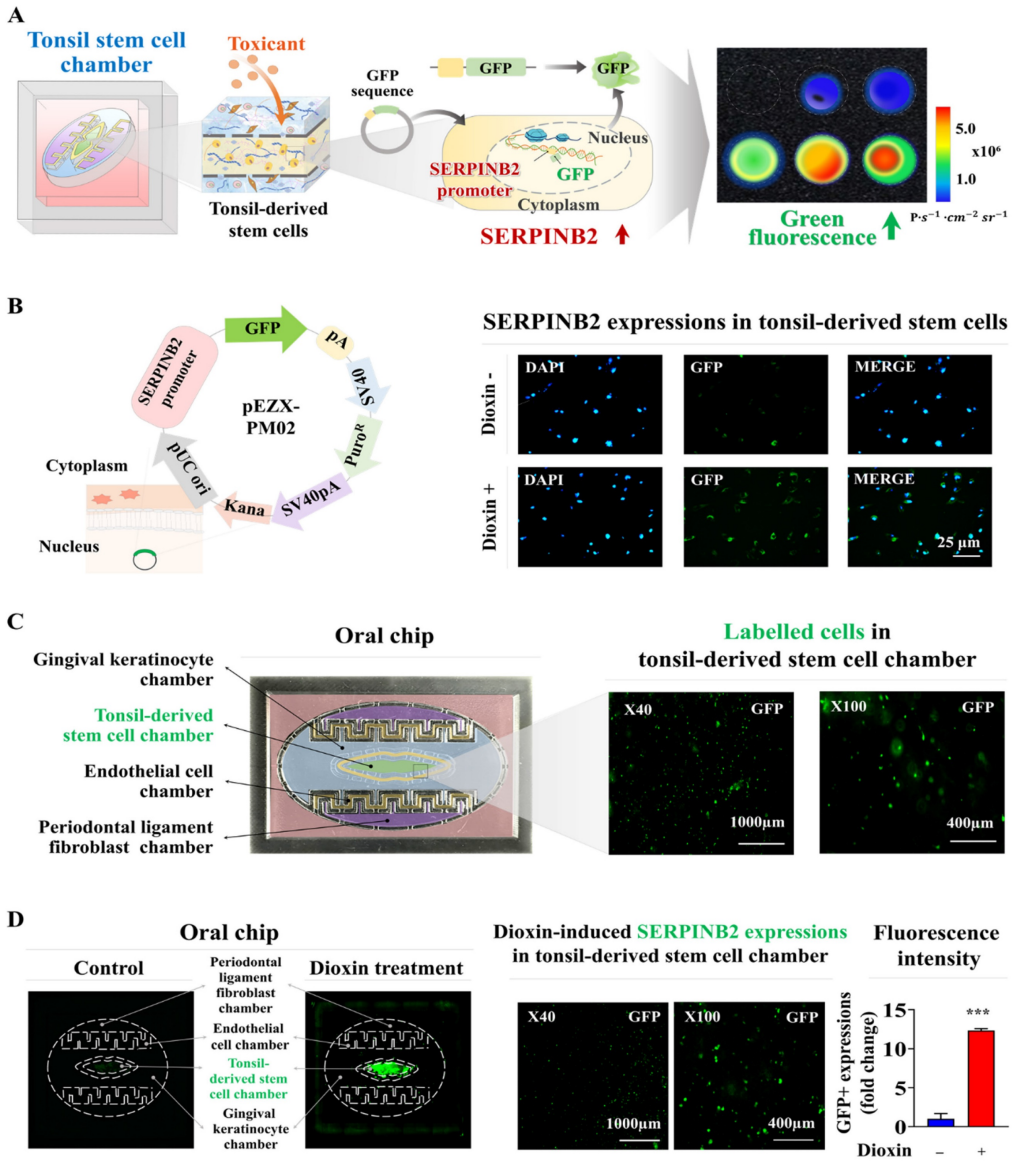
** Development of a fluorescence-based detection platform linked with a toxicity marker within the tonsil stem cell chamber of the oral-on-a-chip model.** A fluorescence toxicity marker detection system equipped with SERPINB2-GFP reporter vector was successfully integrated into human tonsil-derived stem cells. The activation of SERPINB2 by exposure to dioxin was manifest as GFP fluorescence allowing the evaluation of oral toxicity in drug candidates using both qualitative and quantitative analyses by measuring the intensity of the fluorescent signal **(A)**. Human tonsil-derived stem cells were stably transfected with GFP-labeled SERPINB2 reporter vector that produces green fluorescence. After these cells were exposed to 5 ng/ml dioxin, immunostaining revealed the notable increase in SERPINB2 activity, evident as green fluorescence **(B)**. This fluorescence-based detection system was integrated into human tonsil-derived stem cells strategically placed within the tonsil stem cell chamber of the oral-on-a-chip. The spatial arrangement of these cells within the designated chamber was analyzed using the fluorescence imaging system **(C)**. Exposure to 5 ng/ml dioxin significantly increased SERPINB2 activity, clearly evident as green fluorescence in the tonsil stem cell chamber of the chip **(D)**. The data are presented as the mean ± standard deviation. *, *p* < 0.05; **, *p* < 0.005; and ***, *p* < 0.001 (two-sample t-test).

**Figure 9:**
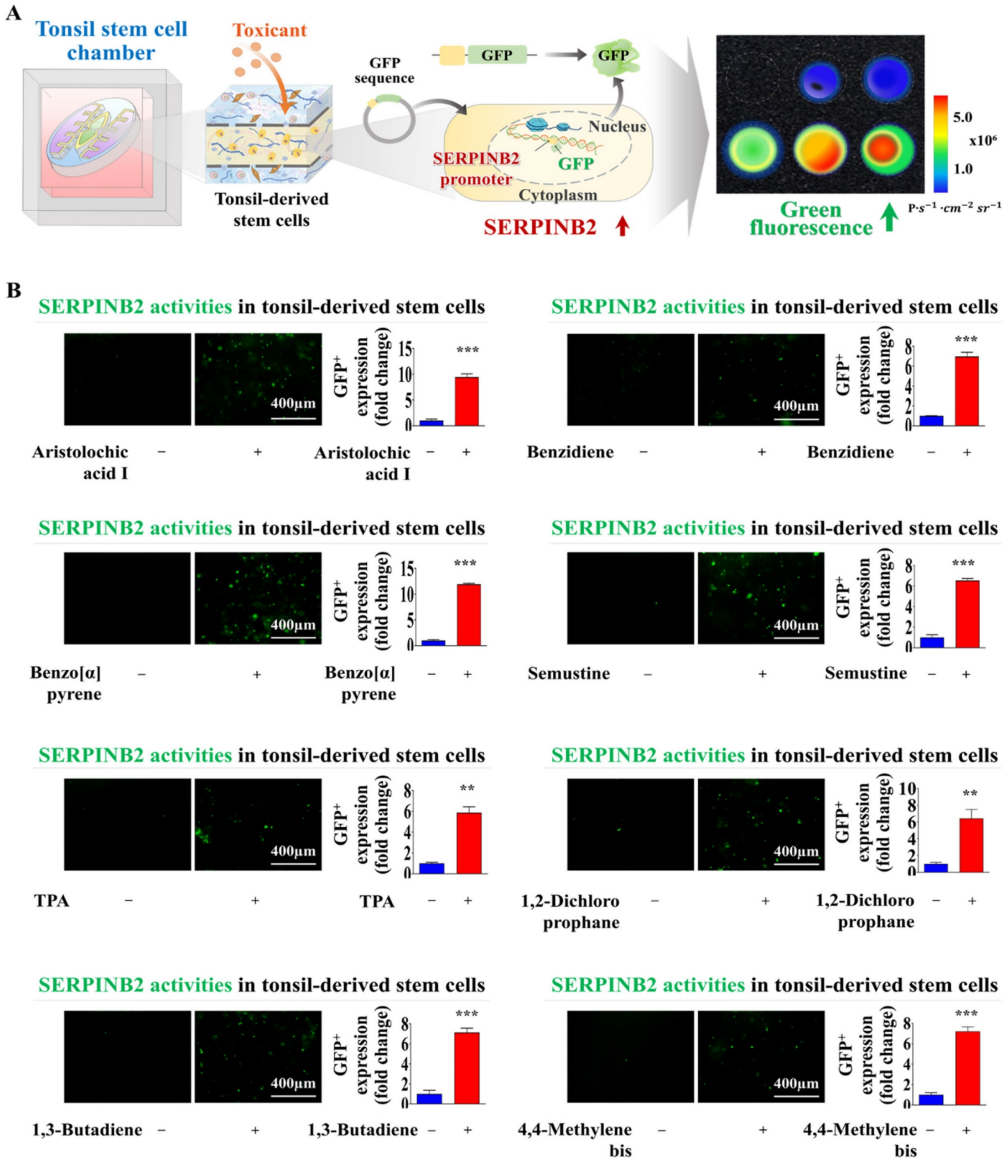
** Exposure to various types of toxicants triggers the activation of SERPINB2, resulting in emission of green fluorescence signal within the tonsil stem cell chamber of the chip.** A detection system utilizing GFP-tagged SERPINB2 reporter was successfully integrated within human tonsil-derived stem cells. These cells were then strategically placed within the tonsil stem cell chamber of the chip platform **(A)**. To evaluate the accuracy and reliability of the SERPINB2-tagged fluorescence detection system, various toxic substances were added to the tonsil stem cell chamber of the chip. These substances included aristolochic acid I (10 μM), benzidine (10 μM), benzo[a]pyrene (2 μM), semustine (0.5 mM), TPA (5 nM), 1,2-dichloropropane (100 mM), 1,3-butadiene (10 mM), and 4,4'-methylenebis (5 μM). The evaluation of the SERPINB2 response to the toxic substances was performed by both qualitative observation and quantitative analysis, measuring the intensity of the emitted SERPINB2-linked green fluorescence signal **(B)**. Significant differences are indicated as follows: *, *p* < 0.05; **, *p* < 0.005; and ***, *p* < 0.001 (two-sample t-test).

Screening platforms linked to fluorescent proteins are widely used to evaluate the functions of particular genes or signaling pathways, providing a reliable tool for determining the potential effects and risks associated with pharmaceutical candidates 39-41. Our results indicated that SERPINB2 functions as a reliable and effective marker for predicting oral toxicity. GFP was incorporated into the promoter region of SERPINB2 in human tonsil stem cells. This configuration enables the activation of SERPINB2 by toxic substances, which in turn triggers a green fluorescent response in the oral cavity of the chip system. This GFP-conjugated toxicity detection system allowed for an easily interpretable and measurable analysis of potential oral toxicity based on the intensity of the fluorescent signals (Fig. 8A). Before embedding the cells containing the fluorescent protein-conjugated SERPINB2 reporter system in the chip platform, the dioxin reference toxicant was applied to evaluate the effectiveness of the toxicity marker-linked fluorescent signal emission (Fig. 8B). Human tonsil-resident stem cells stably transfected with the GFP-conjugated SERPINB2 reporter construct were incorporated into the tonsil chamber of the oral-on-a-chip. These cells were evenly distributed within the tonsil chamber of the oral-on-a-chip device (Fig. 8C). Following the incorporation of cells into the chip, the effectiveness of the GFP-conjugated SERPINB2 detection system in measuring oral toxicity was assessed by measuring GFP fluorescence to determine SERPINB2 expression levels in the tonsil chamber in cells exposed to dioxin (Fig. 8D). To determine whether this detection platform could function as a general screening system for detecting oral toxicity across different toxic substance categories, rather than just the specific dioxin reference toxicant, GFP fluorescence corresponding to SERPINB2 activity was measured in the tonsil chambers both in untreated cells and cells exposed to various toxic substances (Fig. 9A). Each of the tested toxic substances significantly increased the GFP fluorescence linked to SERPINB2 activity in the tonsil chambers of the oral-on-a-chip (Fig. 9B). These findings implicate the novel GFP fluorescence-tagged SERPINB2 detection system as an effective and reliable oral toxicity screening platform for diverse potential therapeutic candidates.

## Discussion

The efficacy and potential side effects of drug candidates are typically evaluated using traditional *in vitro* single-cell monolayer culture models *in vitro* 42. Despite their rapid deployment, simplicity, and adaptability, traditional 2D culture models do not adequately mimic the intricate environments of living tissues 43. Specifically, these models do not encompass the essential interactions between cells and ECM or between cells, which are crucial for tissue functionality 44. Such vital interactions play a key role in preserving the physiological integrity of tissues and influencing their reactions to various stimuli 45. Consequently, these 2D cultures may produce inconsistent and unreliable outcomes, often not reflecting the true *in vivo* responses 46. Considerable progress has been made in developing advanced oral on-chip platforms to overcome the fundamental shortcomings of traditional single-cell 2D culture systems. For instance, Ly *et al.* developed an oral mucosa-on-a-chip that incorporates a top layer of keratinocytes combined with a collagen hydrogel containing fibroblasts interconnected by pores across a network of three microchannels. This innovative design facilitates the accurate evaluation of oral mucosal reactions to dental biomaterials, such as 2-hydroxyethyl methacrylate 7, using a vertically structured microfluidic system. This configuration allows for prolonged culture at the air-liquid interface and replicates the interactions between the host and materials under dynamic flow conditions. This system includes a top-flow component containing human gingival fibroblasts and human oral keratinocytes within a fibrin-based human mucosal matrix, which is precisely engineered to replicate the mechanical impact of mouthwash 5. This model facilitates the assessment of interactions between the host and materials, as well as the transmucosal permeability of oral care products that are relevant to both normal and disease conditions.

Despite efforts with existing oral-on-a-chip systems to emulate human oral tissues' complex structure and active conditions, the systems have several major technical limitations. For example, to precisely mimic oral tissue, it is essential to acquire and sustain a variety of oral cells, such as gingival keratinocytes, tonsil-resident stem cells, vascular endothelial cells, and periodontal ligament fibroblasts. Co-culturing these distinct cell types within a single chip system with their unique cultural needs and behaviors introduces complexity and potential for variability. In addition, maintaining the long-term viability and functionality of these cells within the chip platform is challenging because of the absence of a 3D tissue microenvironment and adequate cellular interactions. We developed an innovative 3D culture platform designated oral-on-a-chip to address this challenge. This sophisticated system combines multiple types of human oral cells, including human gingival keratinocytes, tonsil-resident stem cells, periodontal ligament fibroblasts, and vascular endothelial cells, with a mixture of biodegradable natural polymers (hyaluronic acid and collagen) and blood coagulating proteins (thrombin and fibrinogen) (Fig. 1A). By integrating these cellular components, our chip platform effectively mimics the distinct characteristics and complexities of the oral tissue microenvironment. This integration accurately replicates intricate cellular interactions typical of complex oral tissues, encompassing the dynamic communication between cells and ECM. The resulting conditions closely resemble those *in vivo*, which support sustained cellular viability and functionality. Despite a gradual decrease in cell survival rates, more than 75% of the integrated cells remained viable after 21 days (Fig. 3A-D). Importantly, integrating diverse oral tissue cells into a natural polymer-based 3D microenvironment within the chip platform maintained the expression of specific markers over a prolonged period (Fig. 4A-D).

Achieving consistent and reliable outcomes from drug screening is a crucial challenge requiring further resolution. Several factors affect the consistency of results obtained from primary cultured oral tissue cells. These include variations among donors, number of cell passages, and skill level of the researcher performing cell isolation and cultivation. Significant variability in primary oral cells from various patients may arise from genetic disparities, existing health conditions, and environmental factors. This results in variable reactions under experimental conditions. To overcome the challenges associated with primary cultured cells, we immortalized several types of oral tissue cells using the SV40 large T antigen. These immortalized oral cell types were subsequently incorporated into the fabricated oral-on-a-chip platform. Compared with primary cells, immortalized cells tend to maintain more consistent genetic and phenotypic characteristics through multiple passages 47. Using the aforementioned immortalized cells in the development of an oral on-a-chip provides significant benefits over the use of primary cells. Immortalized cells were assessed for the expression of key markers related to their tissue-specific functions. For example, tonsil-derived stem cells maintained high expression of stemness markers such as CD105 and NANOG, while periodontal ligament fibroblasts retained expression of vimentin and fibronectin. The normal expression patterns of these cell specific markers ensure that these immortalized cells preserved their native properties (Fig. 4A-D). ​These advantages include increased cell proliferation, an extended lifespan, heightened reproducibility and scalability for high-throughput use, fewer ethical issues, and enhanced genetic stability and manipulability. While our current oral-on-a-chip system is based on normal oral cells, the platform's versatility allows for straightforward adaptation to simulate a wide range of oral pathologies. By incorporating disease-specific cells or introducing pathological stimuli, the platform can be tailored to mimic various disease states, including oral cancers, periodontal diseases, and inflammatory conditions such as oral lichen planus and oral mucositis. The ability to replicate these conditions will significantly broaden the platform's clinical relevance by allowing for in-depth studies of disease mechanisms, drug efficacy, and personalized therapeutic responses.

Another pivotal hurdle in the effective development of an oral-on-a-chip is the establishment of a precise screening system capable of quantitatively measuring molecular and physiological alterations triggered by external stimuli. The accurate and consistent quantification of these changes is crucial for effectively evaluating the dynamic reactions of oral tissues under different external stimuli, which in turn improves the functionality and efficacy of the oral-on-a-chip platform. A screening approach based on specific biomarkers (genes) has substantial benefits compared to traditional methods that depend on morphological or functional parameters, such as phenotypic alterations, cell proliferation, and apoptosis 48. Biomarker-based screening platforms enable the rapid and sensitive detection of initial disruptions in cellular activities, including oxidative stress, DNA damage, and inflammatory reactions, facilitating earlier and more precise measurement of toxic effects than methods based on functional parameters, which often require prolonged and intense stimulation to detect changes 8. In the present study, RNA-seq analysis on the human tonsil-derived stem cell-based platform identified SERPINB2 as a reliable biomarker for predicting the oral toxicity of certain substances (Fig. 5A-F). We also confirmed a significant correlation between the exposure to various toxic substances and increased SERPINB2 expression (Supplementary Fig. 5 and Supplementary Fig. 6). Notably, SERPINB2 depletion substantially attenuated the toxic exposure-mediated inhibitory effects on human tonsil-resident stem cells (Fig. 7A-H). Consistent with our results, previous studies have shown that exposure to various toxic substances leads to a significant increase in SERPINB2 expression in various stem cell types, including cancer stem cells 49, human endometrial stem cells 50, and umbilical cord blood stem cells 9. These findings indicate that SERPINB2 is a reliable and effective stem cell-based marker for predicting oral toxicity. Subsequently, GFP was incorporated into the upstream regulatory region of SERPINB2 in tonsil-resident stem cells (Fig. 8B). The levels of GFP-tagged SERPINB2 following exposure to toxic substances were quantitatively evaluated by analyzing the intensity of the emitted fluorescent signals (Fig. 8D). A marked increase in GFP fluorescence associated with SERPINB2 was observed in tonsil-resident stem cells on the oral surface of the chip platform after exposure to diverse toxic substances (Fig. 9). Our toxicity marker-based colorimetric system was specifically designed with scalability in mind. The core mechanism relies on the expression of SERPINB2 as a toxicity biomarker, which is coupled with a quantifiable colorimetric output through a green fluorescent protein (GFP) tagging system. This enables rapid and straightforward detection of toxicity levels based on measurable changes in fluorescence intensity. Importantly, the platform was developed to be compatible with standard microplate formats (e.g., 96-well, 384-well plates), ensuring that the detection and analysis processes can be seamlessly integrated into high-throughput screening workflows that are commonly employed in the pharmaceutical industry.

The platform's ability to mimic the physiological environment of the oral cavity makes it an ideal tool for evaluating the efficacy of drug candidates, particularly in the context of oral diseases such as periodontitis, oral cancers, mucositis, and wound healing. In addition, the oral-on-a-chip platform can also be adapted for personalized medicine by incorporating patient-derived cells into the 3D tissue structure. This personalized approach allows for the testing of therapeutic efficacy in a patient-specific context, providing valuable insights into how individual patients might respond to certain drugs.

## Supplementary Material

Supplementary figures.

## Figures and Tables

**Table 1:** Primer sequences for quantitative RT-PCR.

Human gene	GenBank No.	Direction	Primer sequence
*PPIA*	NM_021130	F	TGCCATCGCCAAGGAGTAG
		R	TGCACAGACGGTCACTCAAA
*C-MYC*	NM_002467	F	AAAGGCCCCCAAGGTAGTTA
		R	GCACAAGAGTTCCGTAGCTG
*KLF4*	NM_001314052	F	GAACTGACCAGGCACTACCG
		R	TTCTGGCAGTGTGGGTCATA
*NANOG*	NM_024865	F	TGGGATTTACAGGCGTGAGC
		R	AAGCAAAGCCTCCCAATCCC
*OCT4*	NM_002701	F	AGCCCTCATTTCACCAGGCC
		R	TGGGACTCCTCCGGGTTTTG
*SOX2*	NM_003106	F	AAATGGGAGGGGTGCAAAAGAGGAG
		R	CAGCTGTCATTTGCTGTGGGTGATG
*SERPINB2*	NM_001143818	F	ACCCCCATGACTCCAGAGAACT
		R	GAGAGCGGAAGGATGAATGGAT
*OASL1 v1*	NM_001261825.2	F	TCGTGAACCCTTATGAGCCC
		R	GGAACCTGGAAGGACAGACG
*TMC2 v1*	XM_005260660.5	F	CTATATCTCGGCCCCCTGGA
		R	TGACCTCCACGGATGAGTCT
*SST v1*	NM_001048.4	F	ACCCAACCAGACGGAGAATG
		R	TAGCCGGGTTTGAGTTAGCA
*PCSK2 v1*	NM_002594.5	F	TGCCGAAGCAAGTTACGACT
		R	AACTTCTCCTGCACATCGGG
CCL8 v1	NM_005623.3	F	CAATGTCCCAAGGAAGCTGTG
		R	GGAATCCCTGACCCATCTCTC
EDRNB v2	NM_001201397.1	F	AGCAGAGACCCCGAGCAAAC
		R	TCCGGCTCTGACGAAACGC
CXCL1 v1	NM_001511.4	F	GGAAAGCTTGCCTCAATCCTG
		R	TTGTCACTGTTCAGCATCTTTTCG

## Abbreviations

3D: three-dimensional
CAD: computer-aided design
CCK-8: cell counting kit - 8
DMEM: dulbecco's modified Eagle's medium
ECM: extracellular matrix
FBS: fetal bovine serum
GEO: gene expression omnibus
GFP: green fluorescent protein
HUVECs: Human umbilical vein endothelial cells
KEGG: Kyoyo Encyclopedia of Genes and genomes
KLF4: krüppel-like factor 4
MMP-2/9: matrix metalloproteinase-2
OCT4: octamer-binding transcription factor 4
PDMS: polydimethylsiloxane
PLA: polylactic acid
SEM: scanning electron microscopy
SERPINB2: plasminogen activator inhibitor-2
SOX2: sex-determining region Y-box 2
TBS-T: tris-buffered saline containing Tween-20
