# Supplementary material for: Development of Advanced Oral-on-a-Chip: Replicating the Intricate Human Oral Microenvironment
Source: Int J Biol Sci. 2024 Oct 28;20(15):5888–909. doi: 10.7150/ijbs.104351 (PMC11628345; doi:10.7150/ijbs.104351)
Supplement: Supplementary file 1 — Supplementary figures. [file ijbsv20p5888s1.pdf]

Supplementary Figs and legends

Supplementary figure 1

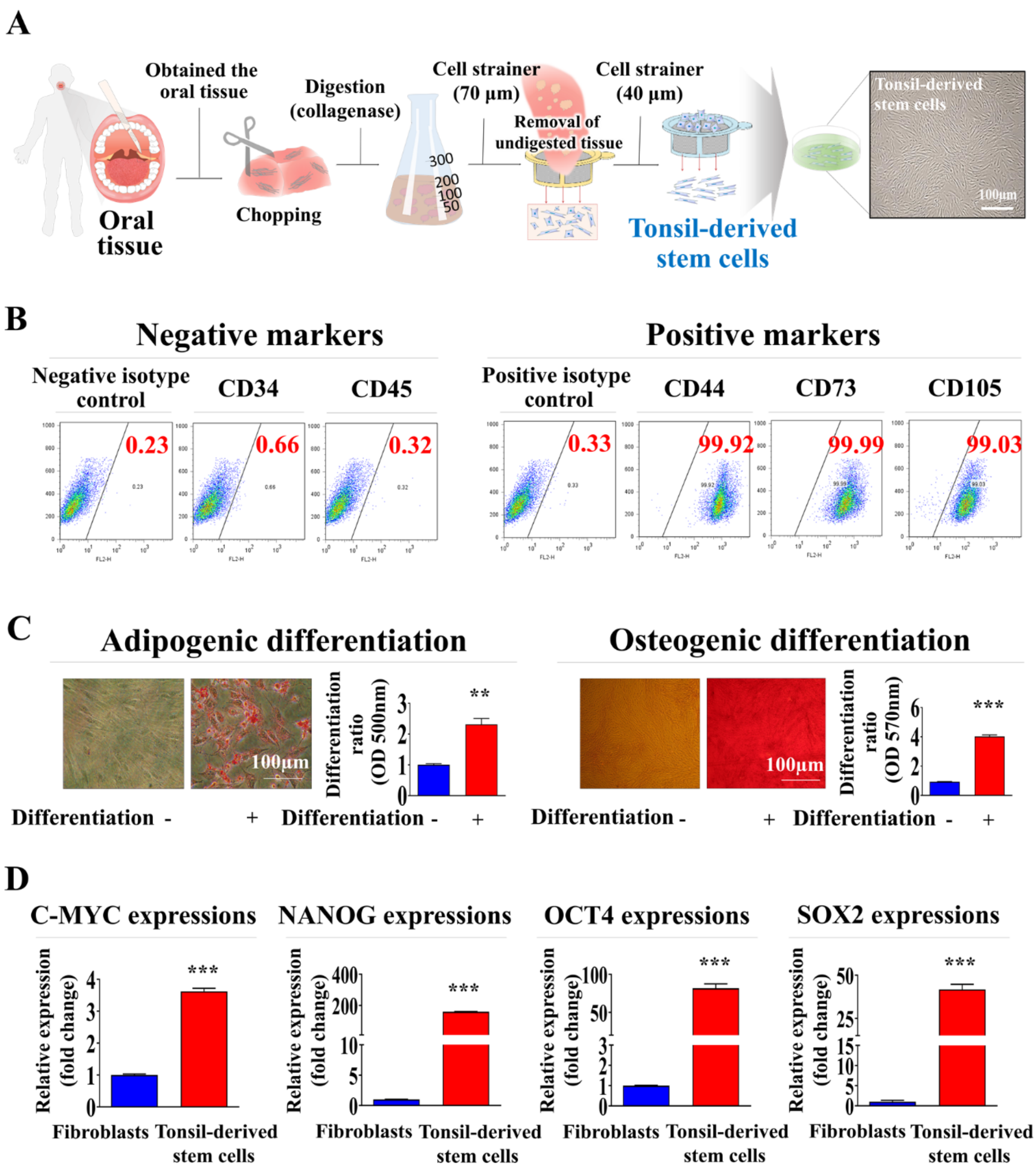

Supplementary Fig. 1. Isolation and characterization of tonsil-derived stem cells from human tonsil tissues. Tonsil tissues were meticulously chopped into fine pieces and digested enzymatically with type I collagenase to isolate stem cells. The extracted stem cells were examined for their morphological characteristics by inverted phase-contrast microscopy **(A)**. Flow cytometry utilizing a

specific set of antibodies was also performed to identify a variety of stem cell markers, including CD44, CD73, and CD105. Additionally, antibodies against hematopoietic markers CD34 and CD45 were used to ensure detailed characterization of the cells **(B)**. The potential of tonsil-derived stem cells to differentiate into adipocytes and osteoblasts was assessed by staining with Oil Red O Alizarin Red S, respectively **(C)**. Mineralization (calcium deposition) and lipid droplet accumulation in differentiated cells was measured at a wavelength of 500 and 570 nm, respectively. The gene expression levels of C-MYC, NANOG, OCT4, and SOX2 pluripotency markers in cells was assessed using real-time PCR **(D)**. All experiments were performed in triplicate. Significant differences are indicated as follows: *, $p < 0.05$; **, $p < 0.005$; and ***, $p < 0.001$ (two-sample t-test).

Supplementary figure 2

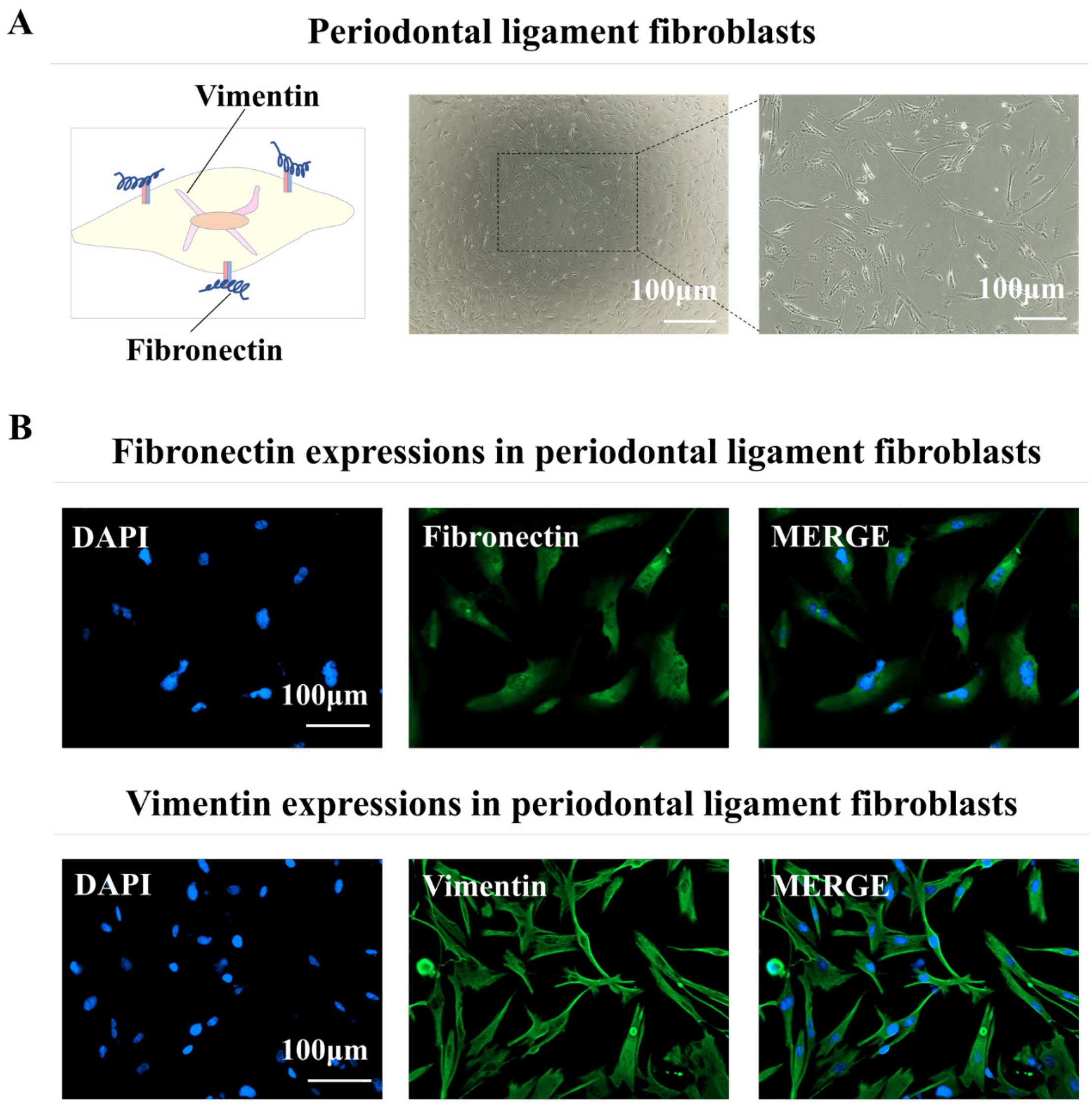

Supplementary Fig. 2. Isolation and molecular characterization of isolated human periodontal ligament fibroblasts. Periodontal ligament tissues were isolated from patients receiving orthodontic procedures. The process included the careful selection of fibroblasts from oral tissue samples using cell strainers of various pore sizes to ensure the purity of the isolated cells. Optical phase microscopy was used to observe the distinctive spindle-shaped morphology typical of human periodontal ligament fibroblasts **(A)**. Immunohistochemical techniques were used to evaluate the presence of key biomarkers such as fibronectin and vimentin in periodontal ligament fibroblasts, revealing their expression profiles **(B)**. DAPI staining was used to label the nuclei within each field.

Supplementary figure 3

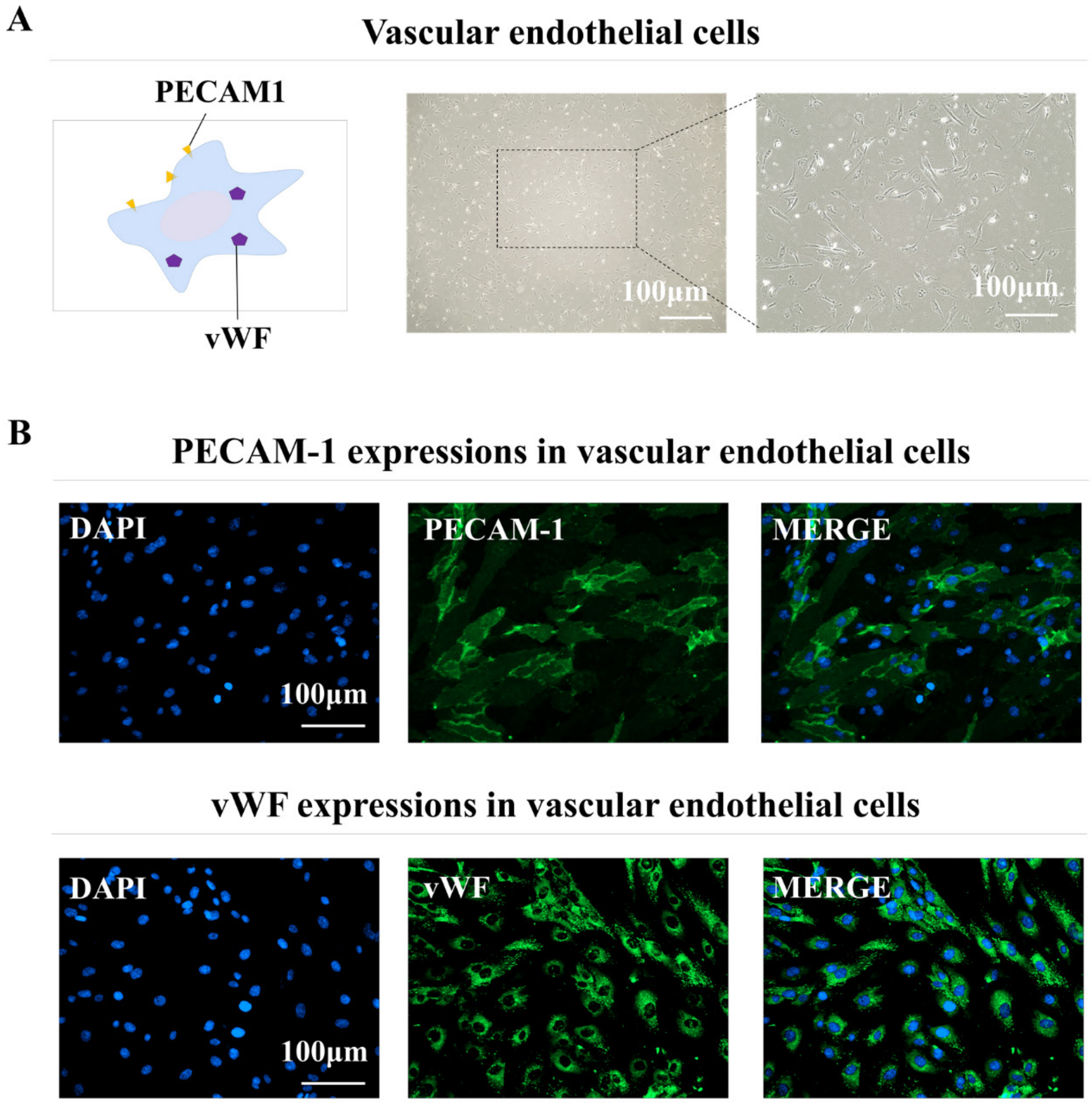

Supplementary Fig. 3. Molecular profile analysis of human umbilical vascular endothelial cells (HUVECs). HUVECs were cultured to form a monolayer and observed by phase-contrast microscopy to confirm their polygonal shape **(A)**. The cells were then subjected to fluorescent immunostaining to evaluate the expression of the specific endothelial markers platelet endothelial cell adhesion molecule-1 (PECAM-1) and von Willebrand Factor (vWF) (**B**). DAPI staining was used to label the nuclei within each field.

Supplementary figure 4

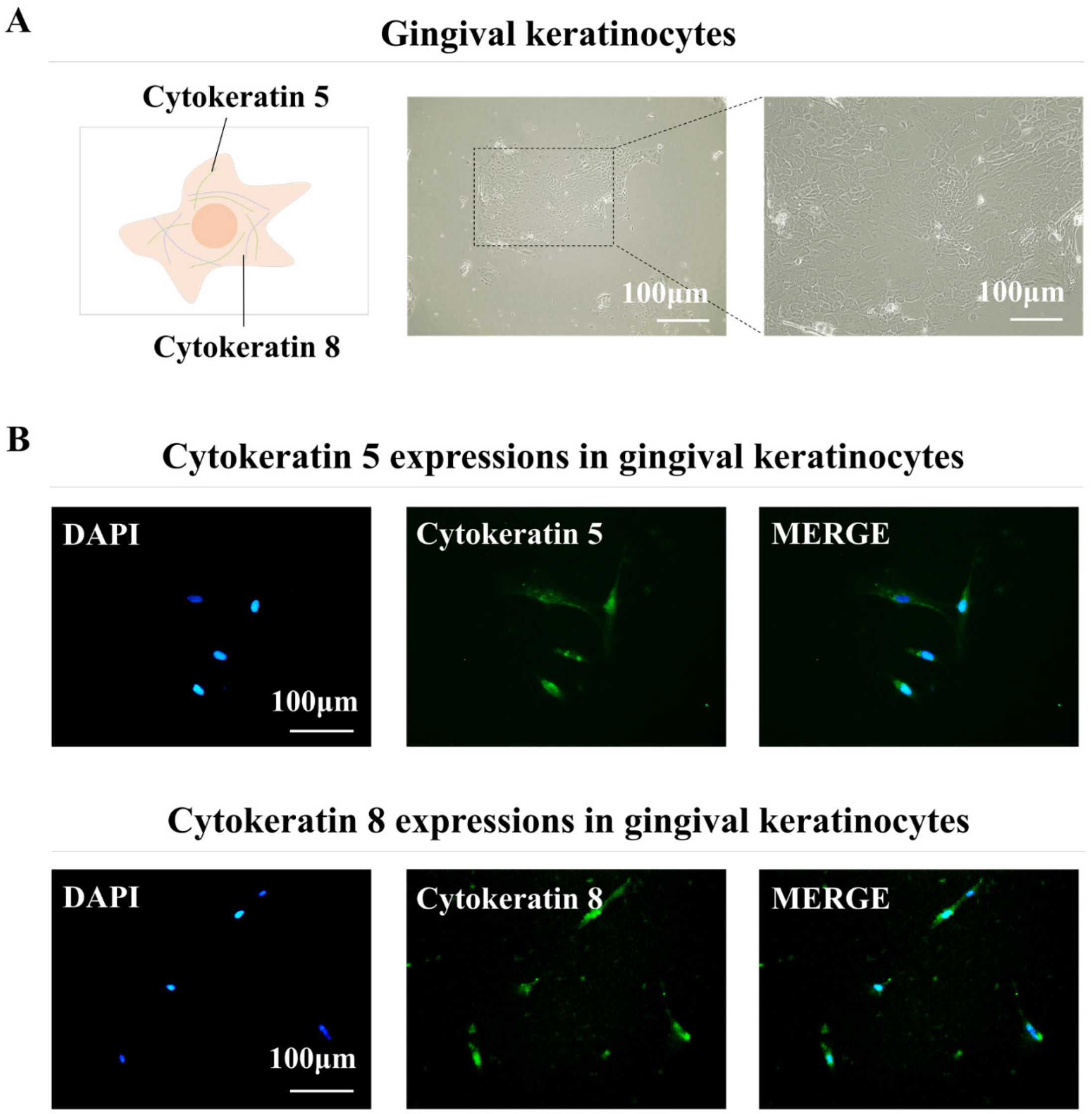

Supplementary Fig. 4. Molecular profile analysis of human gingival keratinocytes. Human gingival keratinocytes were identified by their unique polygonal structure **(A)**. The presence of the key cytokeratin 5 and 8 biomarkers was examined in these cells by fluorescence immunostaining **(B)**. DAPI staining was used to label the nuclei within each field.

Supplementary figure 5

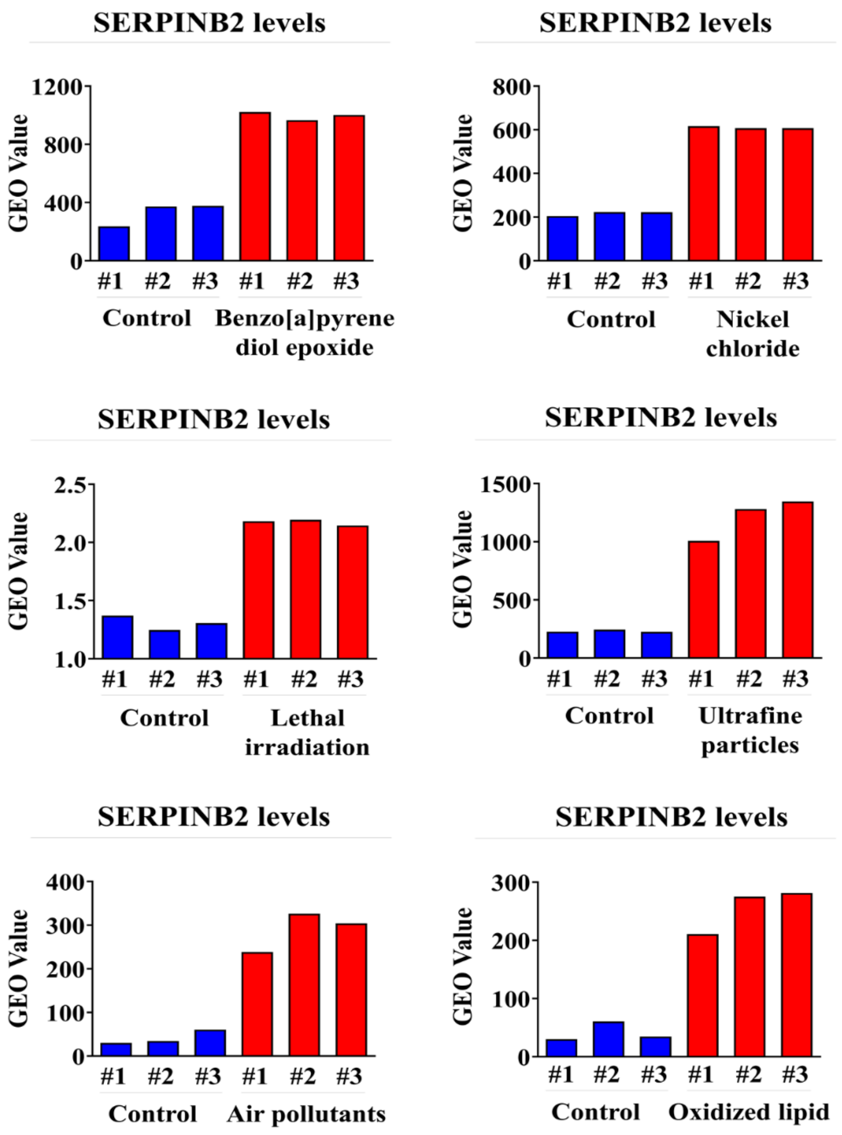

Supplementary Fig. 5. Analysis of public datasets to evaluate the expression of SERPINB2 following exposure to various toxicants. Data from the Gene Expression Omnibus (GEO) database revealed significant correlations between increased SERPINB2 expression and exposure to benzo[a]pyrenediol epoxide, nickel chloride, lethal irradiation, ultrafine particles, and oxidized lipids.

Supplementary figure 6

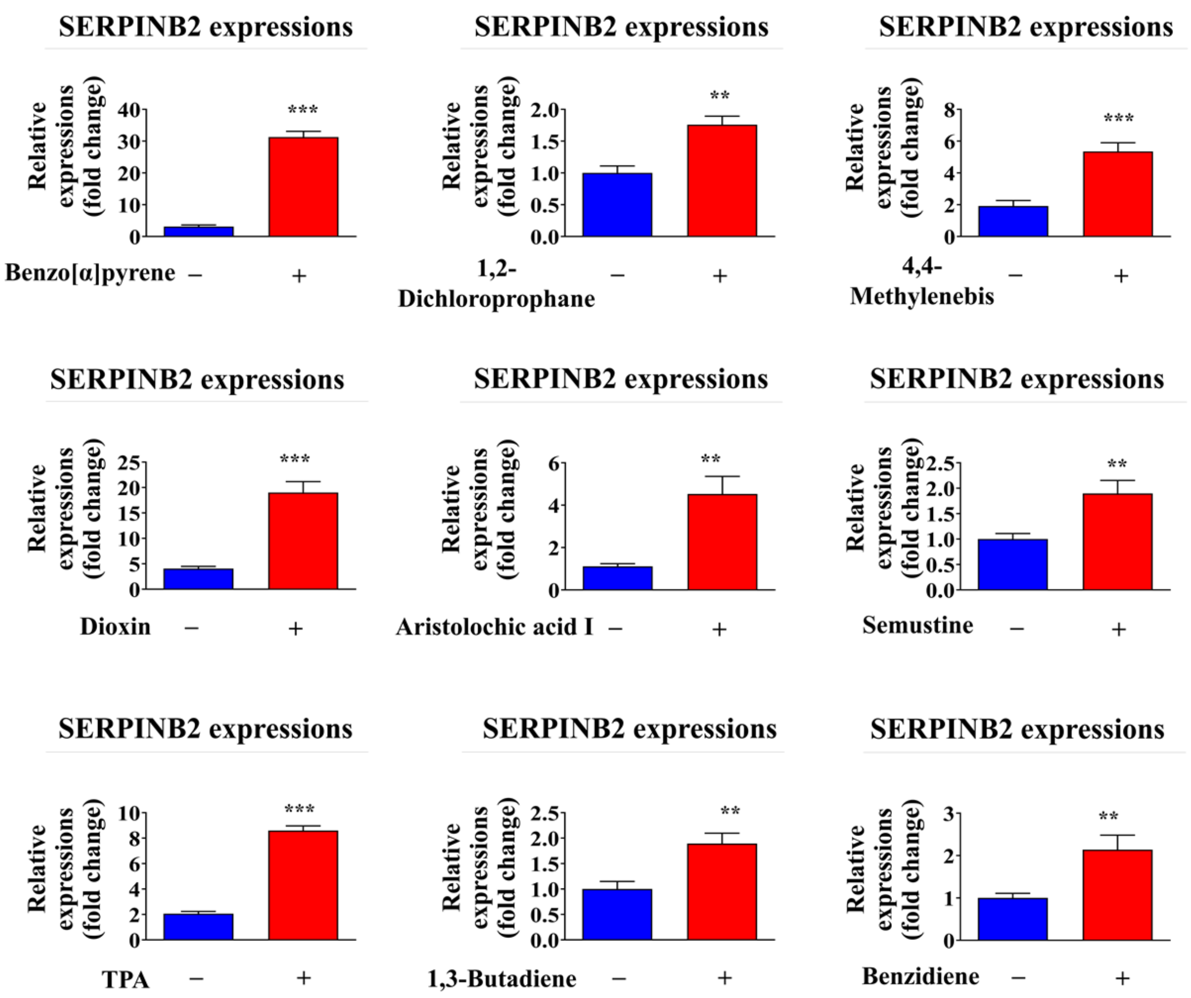

Supplementary Fig. 6. Exposure to various toxicants significantly increases SERPINB2 expression in stem cells derived from human tonsils. Quantitative PCR was utilized to measure SERPINB2 gene expression in human tonsil-derived stem cells following 72 h exposures to benzo[a]pyrene, 1,2-dichloropropane, 4,4'-methylenebis, dioxin, aristolochic acid I, semustine, TPA, 1,3-butadiene, and benzidine at specified concentrations. *Peptidyl-prolyl cis-trans isomerase* (*PPIA*) was used as a housekeeping gene for real-time PCR. Significant differences are presented as *, $p < 0.05$; **, $p < 0.005$; and ***, $p < 0.001$ (two-sample t-test).

Supplementary figure 7

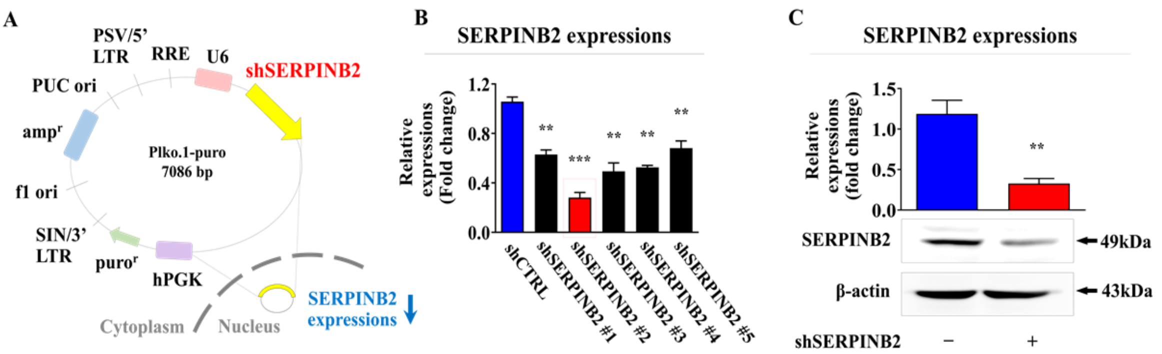

Supplementary Fig. 7. Investigation of the effectiveness of specific shRNAs targeting SERPINB2 in reducing its expression in human tonsil-derived stem cells. Human tonsil-derived stem cells were successfully transfected with a series of shRNA constructs (#1 to #5) aimed at targeting SERPINB2 **(A)**. Construct #2, later identified specifically as SERPINB2 shRNA, demonstrated the highest efficacy in diminishing SERPINB2 at both the mRNA **(B)** and protein **(C)** levels. β-actin was used as an internal protein control. *PPIA* was used as a housekeeping gene for real-time PCR. All experiments were performed in triplicate. Data are presented as mean ± standard deviation. *, $p < 0.05$; **, $p < 0.005$; and ***, $p < 0.001$ (two-sample t-test).
